# Supplementary figures and images for: Trial-by-trial predictions of subjective time from human brain activity
Source: PLoS Comput Biol. 2022 Jul 7;18(7):e1010223. doi: 10.1371/journal.pcbi.1010223 (PMC9262235; doi:10.1371/journal.pcbi.1010223)

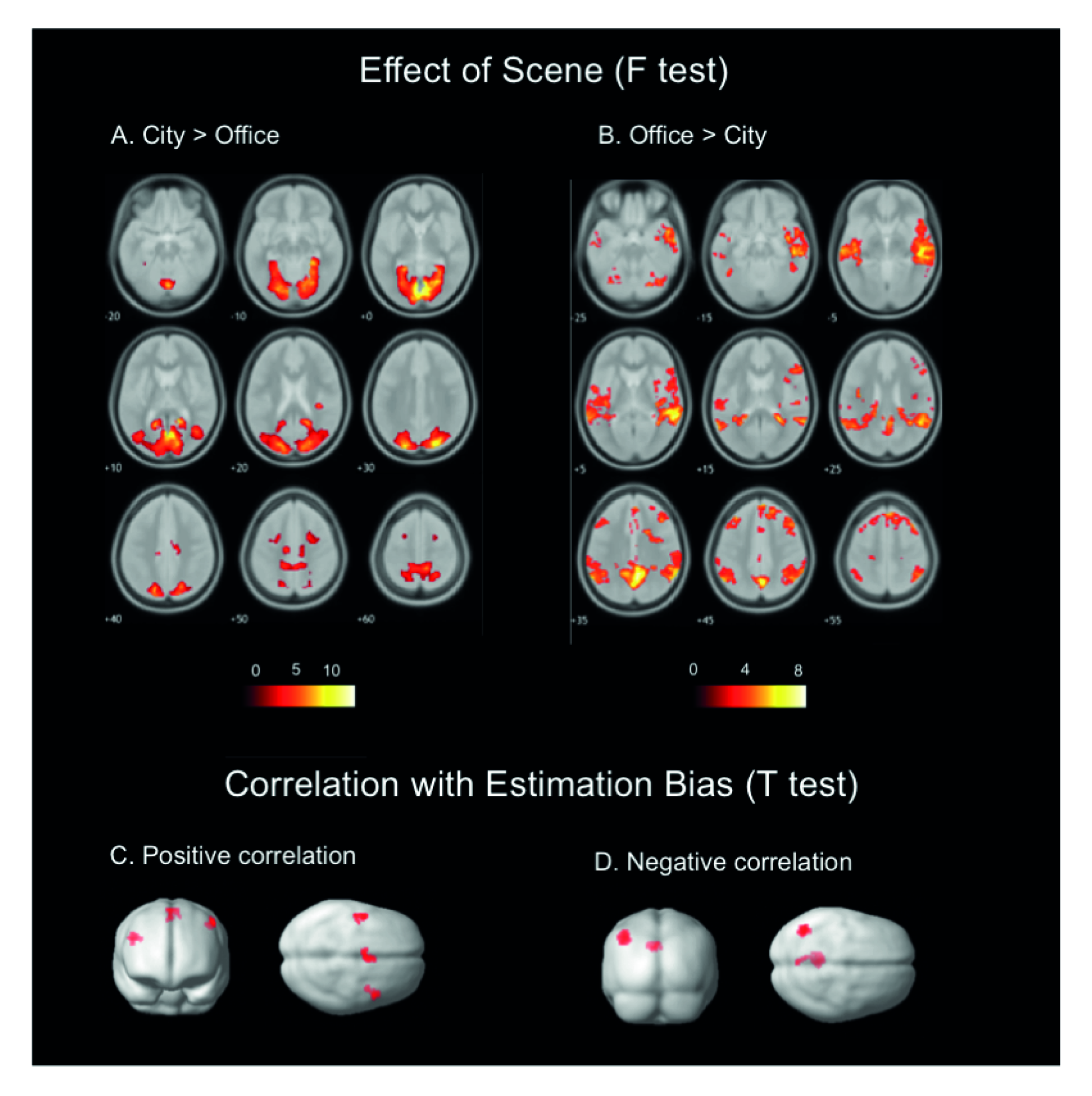

Supplement: S1 Fig — A Higher BOLD for city than office scenes: R lingual gyrus; bilateral midcingulate area; R insula; bilateral SFG. B Higher BOLD for office than city scenes: R precuneus; bilateral precentral gyrus; L MFG; bilateral cerebellum; L paracentral lobule; R SFG. C Positive correlation with normalized estimation bias: bilateral precentral gyrus; L SMA; R superior occipital gyrus. D Negative correlation with normalized estimation bias: L angular frontal gyrus; L MFG; L posterior cingulate. See also S2 Table. (TIF) [file pcbi.1010223.s001.tif]

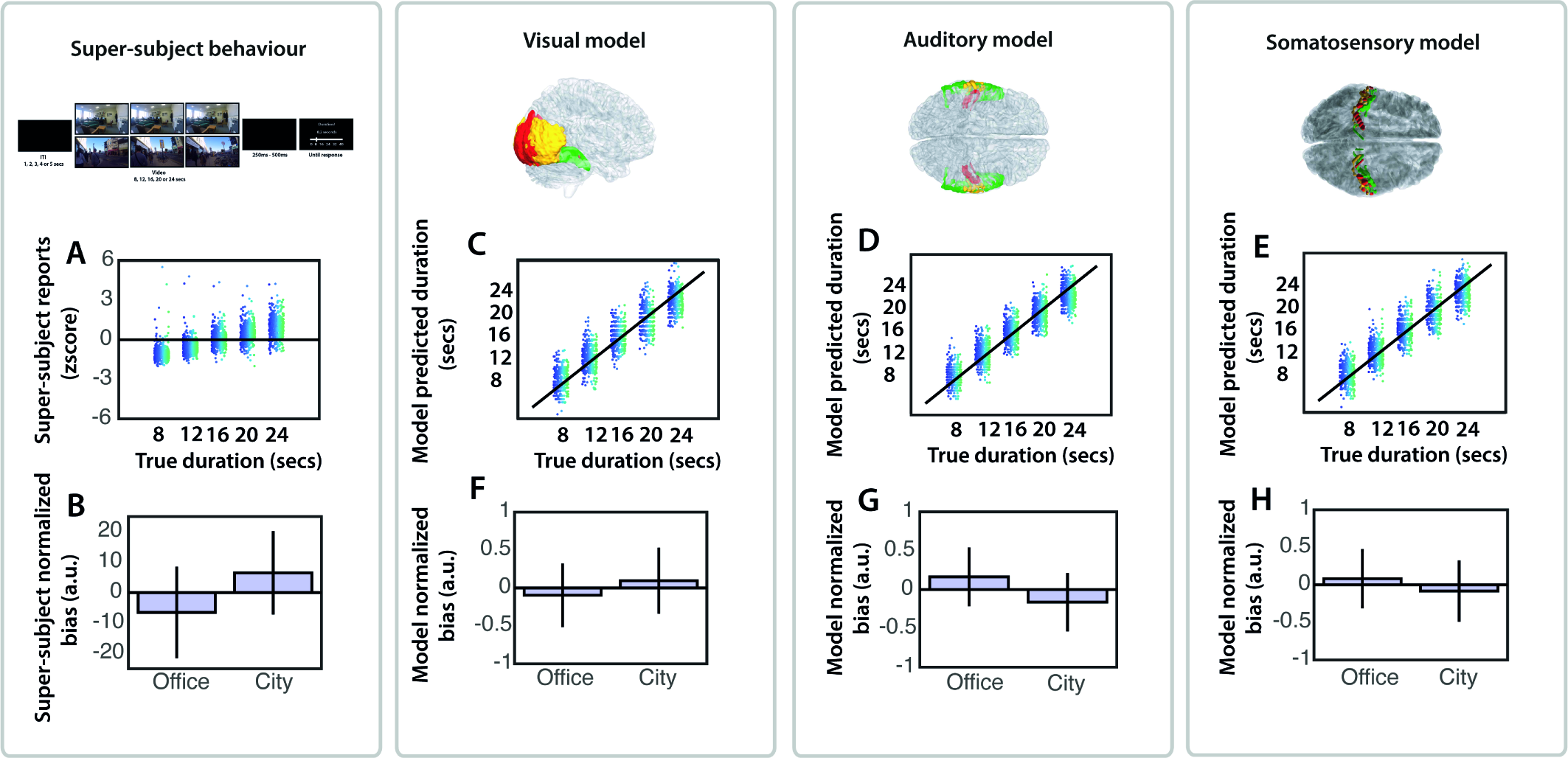

Supplement: S2 Fig — (A) Strong positive association between presented video durations and the z-scored reports we used to build the super-subject. (B) Normalized estimation bias computed on pooled (‘super-subject’) behavioral data, as a function of video scene. (C-E) Association between presented video duration and model-predicted durations separately for visual, auditory and somatosensory Euclidean Distance models respectively. (F-H) Mean normalized bias of the visual, auditory and somatosensory models respectively, for office versus city scenes. Dot colors in the scatterplots represent different participants. Error bars in the bar charts represent SEM. (TIF) [file pcbi.1010223.s002.tif]

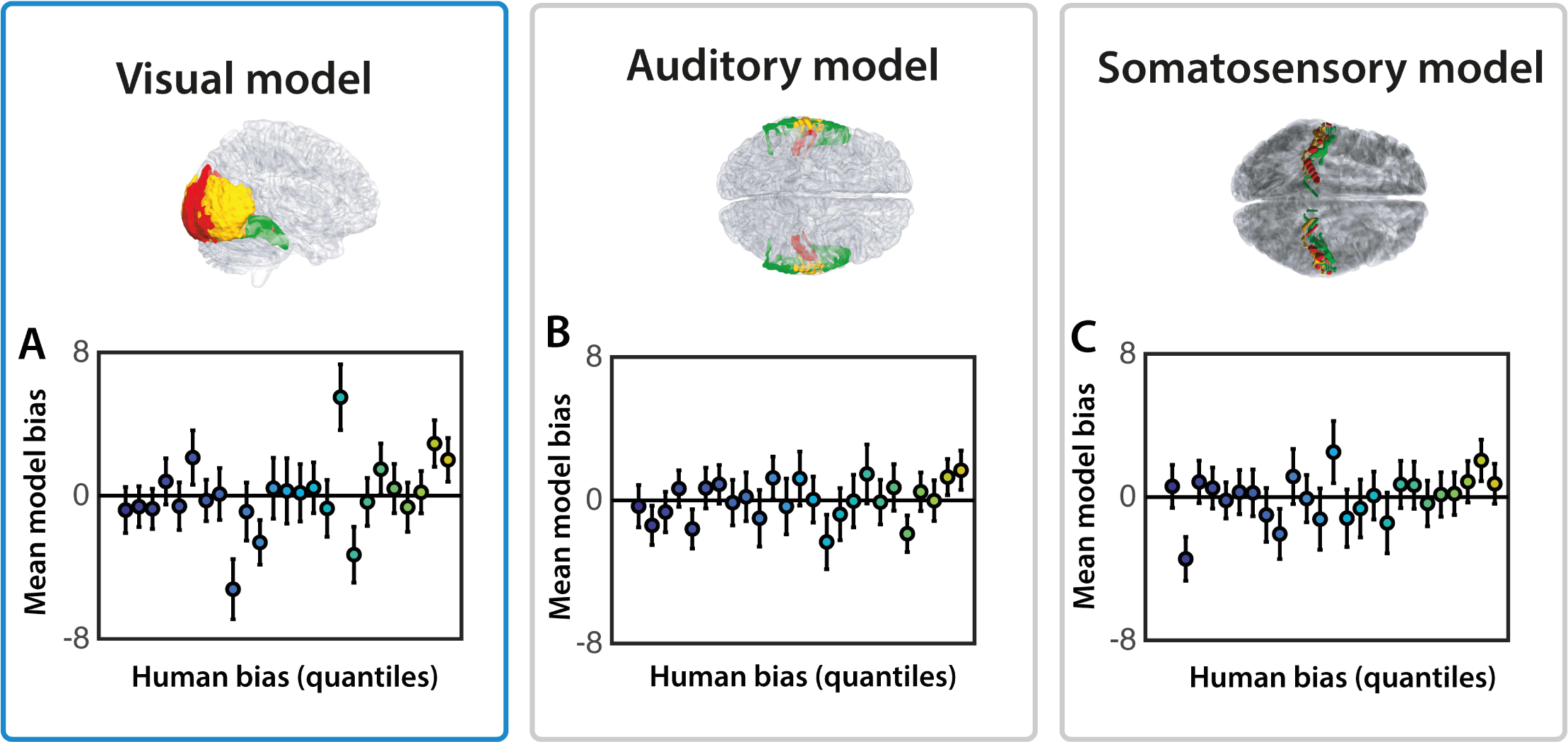

Supplement: S3 Fig — Normalized bias predicted by models trained on salient events (Euclidean distance) in (A) visual, (B) auditory and (C) somatosensory hierarchies. On the x-axis is the 25 bins representing 25 quantiles of human super-subject bias, and on the y-axis is mean model bias for the trials that fell within in the respective bins. Error bars represent +/- SEM. (TIF) [file pcbi.1010223.s003.tif]

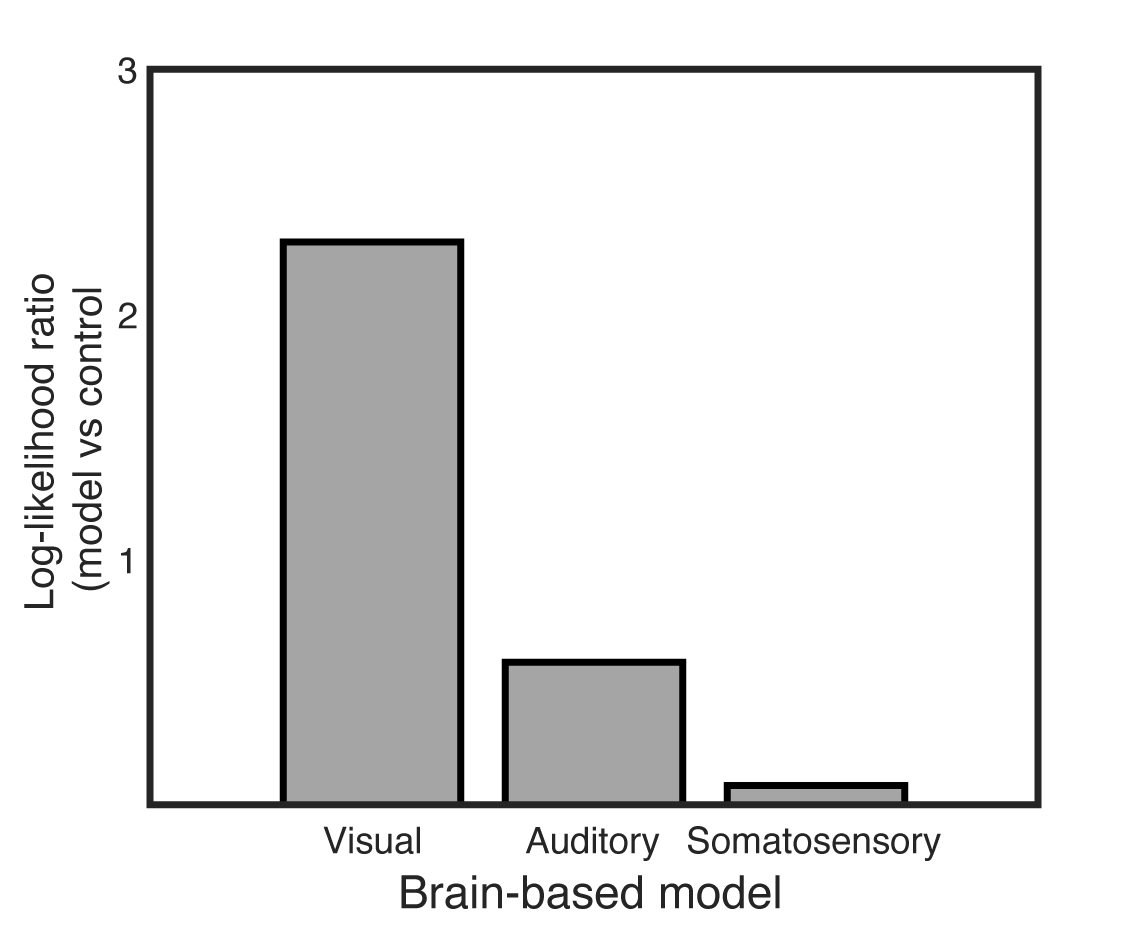

Supplement: S4 Fig — To compare the performance of the three regressions we compared their log-likelihoods to the null (intercept) model (higher values indicate better model fits). The visual cortex regression outperforms the other two, as indicated by its higher log-likelihood ratio. (TIF) [file pcbi.1010223.s004.tif]

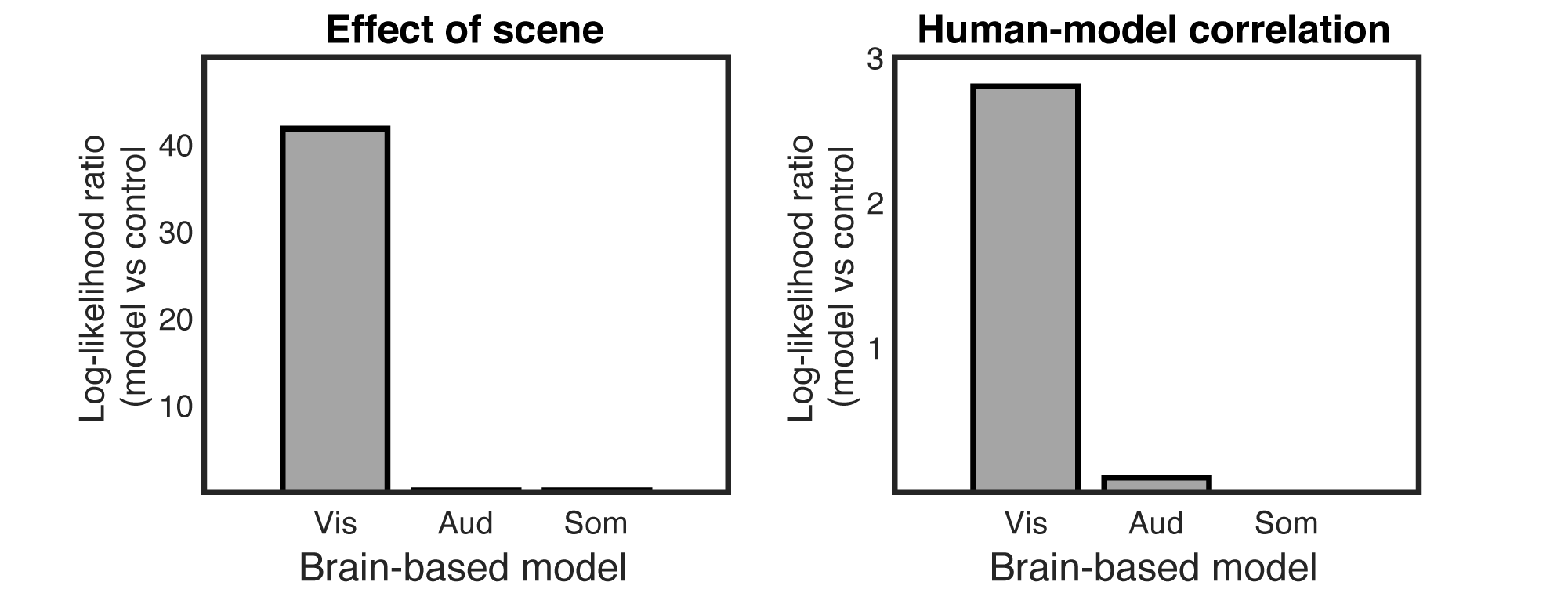

Supplement: S5 Fig — Left. To test whether the visual, auditory or somatosensory models generated predicted durations that discriminated video type, we ran linear mixed models (LMMs) predicting model biases from the fixed effect video scene (city vs office). These were compared to control LMMs that did not have this fixed effect, using the log-likelihood ratio (LLR). The visual cortex LMM outperformed the auditory and somatosensory cortex LMMs as indicated by the greater LLR. Right. For each of the visual, auditory and somatosensory models, we constructed an LMM with human bias as the outcome and the model-predicted biases as a fixed effect. These LMMs tested the video-by-video correlations between predicted and human bias. These LMMs were compared to control models that did not have the model-predicted bias as a fixed effect, using LLR. The visual cortex LMM outperformed the auditory and somatosensory cortex LMMs, as indicated by the greater LLR. (TIF) [file pcbi.1010223.s005.tif]

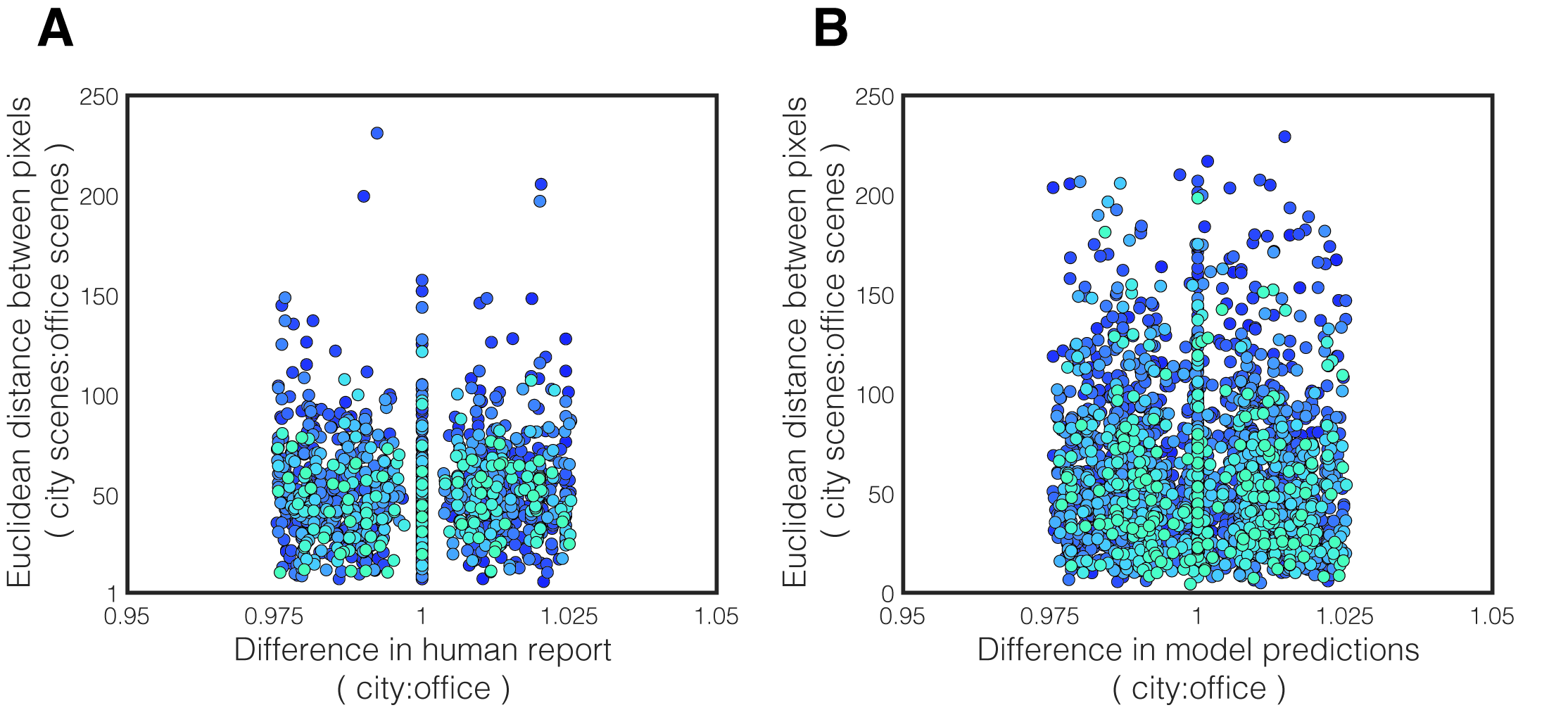

Supplement: S6 Fig — We identified pairs of trials from the same participant, but from different video categories for which (A) human reports were very similar (the log ratio did not exceed 0.025) or (B) the reports predicted by the visual cortex model were very similar (the log ratio did not exceed 0.025). In both panels A and B, a dot represents a pair of trials. Dot colour represents the participant. For each pair, differences in report (human in panel A or model-predicted in panel B) are plotted against differences in the physical video differences, here quantified as the frame-to-frame Euclidean distance averaged over pixels. The difference in report/Euclidean distance between the two trials in a pair is expressed as log(city/office). These figures show that there were many trials pairs in our data where, despite being very different in terms of the pixel differences (up to 100s of times), human duration estimations (A) and visual cortex-based model predictions (B) were almost identical. (TIF) [file pcbi.1010223.s006.tif]
