# Supplementary material for: Trial-by-trial predictions of subjective time from human brain activity
Source: PLoS Comput Biol. 2022 Jul 7;18(7):e1010223. doi: 10.1371/journal.pcbi.1010223 (PMC9262235; doi:10.1371/journal.pcbi.1010223)
Supplement: S1 Text — Pixel-wise changes in stimulation are dissociable from both human and model-predicted report. (DOC) [file pcbi.1010223.s007.doc]

**Supplementary Results**

**Pixel-wise changes in stimulation are dissociable from both human and model-predicted report**

It might be suggested that because we used naturalistic video stimuli rather than simplified, abstract stimuli, our approach is confounded by basic stimulus properties – perhaps only the low-level stimulus change present in the stimuli is driving participant and model estimation. However, attempting to model human time perception based on only one type of stimulus change at a single level is unlikely to work for the reasons mentioned in the Introduction, namely that changes at higher or lower-levels of the processing hierarchy can be seen to contribute to distortions in time perception.

To further assuage this worry in the present study, we examined pairs of trials where the presented scene type differed (city vs office), and where the physical difference in pixel-wise changes in the videos differed greatly (up to 231 times more physical change in a city than office scene), despite reported human durations being almost identical (within 2.5% of each other). For each video clip presented to participants, we calculated the mean physical frame-by-frame change in each video as:


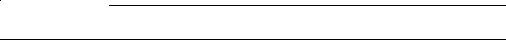


where *t* is a frame, each pixel is an (R,G,B) triplet of intensity values, and there are k_x_ x k_y_ pixels. This equation simply states that we calculated frame-to-frame Euclidean distance for each pixel separately, then summed over all pixels and timepoints. For interpretability, we divided by the number of frames so that longer videos didn’t necessarily take higher values.

To identify trials where experienced durations were similar despite differing physical stimulation, we took all pairs of trials where the following conditions were met:

1. Both trials came from the same participant
2. The pair consisted of one office and one city scene
3. The log ratio between the reported durations for the two videos did not exceed 0.025, i.e. |log(report_1_/report_2_)| <= 0.025 was satisfied.

As can be seen in S6 Fig, there were many instances in our data where ~~the~~ basic stimulus properties (here, pixel intensity) changed at a vastly higher rate in one video than another (100s of times more change in some city videos versus office videos) yet participants reported these videos as being of a very similar length (note the very dense distribution of x-axis values). Despite there being clear overlap in duration reports for many instances of the different scene types, there was clearly an overall difference in duration estimation by scene (see Fig 1C). We repeated the above process exactly but seeking trials that differed in the visual cortex model predictions instead. The results are plotted in S6B Fig, and again, show that two trials with substantially different stimulus properties can nonetheless be associated with similar duration estimates predicted from visual cortex BOLD.

These results are what one would expect given that the stimuli we used are naturalistic videos: namely, there is a wide distribution of physical changes at the most basic level because the videos contain real scenes filmed in the world, rather than abstract stimuli typically used in studies of time perception. These basic physical changes are associated with different types of natural events – a bus going past on the adjacent street, a person walking in front of the camera, a person appearing in view in a largely empty office – but not through a direct one-to-one mapping.

These two results demonstrate that the basic stimulus properties (here, simplified as change in pixel intensity) can dissociate from behavioural response. This may seem to be a trivial point, but it is important for highlighting the need to consider internal responses (as indicated by brain activity, measured or modelled). We are not claiming that changes in low-level stimulus features are irrelevant for time perception – of course, much of perceptual processing will be stimulus-driven. However, we do not take the approach that perceptual experience of these visual scenes directly and precisely reflects the physical qualities of those scenes without change; rather, in order to explain perceptual experience we need to consider neural responses to stimulation (modelled or measured), and not just the stimulation itself. That our approach tracks changes in brain dynamics across a hierarchy of processing is a feature that allows the model to deal with time perception on for natural scenes. It is not a confound to be eliminated by considering only video stimuli perfectly matched for their lowest-level properties (i.e, mean pixel-wise distance) – since doing so would render such stimuli uninformative about naturalistic perception.
