# Supplementary material for: Trial-by-trial predictions of subjective time from human brain activity
Source: PLoS Comput Biol. 2022 Jul 7;18(7):e1010223. doi: 10.1371/journal.pcbi.1010223 (PMC9262235; doi:10.1371/journal.pcbi.1010223)
Supplement: S1 Table — (PDF) [file pcbi.1010223.s008.pdf]

S1 Table. Definition of hierarchies for each sensory cortex model

	Visual	Auditory	Somatosensory
Layer 1	V1, V2v, V3v	BA41	BA3
Layer 2	hV4, LO1, LO2	BA42	BA1
Layer 3	VO1, VO2, PHC1, PHC2	BA22	BA2
