# Supplementary material for: Trial-by-trial predictions of subjective time from human brain activity
Source: PLoS Comput Biol. 2022 Jul 7;18(7):e1010223. doi: 10.1371/journal.pcbi.1010223 (PMC9262235; doi:10.1371/journal.pcbi.1010223)
Supplement: S2 Table — (PDF) [file pcbi.1010223.s009.pdf]

**S2 Table.** Criterion parameters for each hierarchical layer of the sensory cortex models

| **Layer** | $a$ | $\vartheta_{max}$ (SD above the mean) | $\vartheta_{min}$ (SD below the mean) |
|---|---|---|---|
| **1** | 0.5 | 0.5 | 1 |
| **2** | 1 | 1 | 0.5 |
| **3** | 1.5 | 1.5 | 0 |
