# Supplementary material for: Trial-by-trial predictions of subjective time from human brain activity
Source: PLoS Comput Biol. 2022 Jul 7;18(7):e1010223. doi: 10.1371/journal.pcbi.1010223 (PMC9262235; doi:10.1371/journal.pcbi.1010223)
Supplement: S3 Table — (PDF) [file pcbi.1010223.s010.pdf]

S3 Table. Criterion parameters for the artificial network model

Layer	t_{max}	t_{min}
conv1	39973	0
conv2	11601	0
conv3	5515	0
conv4	3244	0
conv5	1117	0
fc6	124	0
fc7	31	0
output	0.33	0

For all layers, $\alpha = 0.001/t_{max}$, $\tau = 6.6T_{max}$ while training and $\tau = 10T_{max}$ while testing.
