# Supplementary material for: Trial-by-trial predictions of subjective time from human brain activity
Source: PLoS Comput Biol. 2022 Jul 7;18(7):e1010223. doi: 10.1371/journal.pcbi.1010223 (PMC9262235; doi:10.1371/journal.pcbi.1010223)
Supplement: S4 Table — (PDF) [file pcbi.1010223.s011.pdf]

S4 Table. Significant clusters revealed by confirmatory GLM on BOLD

Region	Size (cm^3)	*T or F*	P_{FWE}	Peak MNI		
				x	y	z
**City > Office (two-tailed)**						
R Lingual Gyrus	121.37	14.69	< 0.001	6	-70	4
L Midcingulate Area	1.20	8.86	0.002	-10	-20	46
R Insula	0.6	7.91	0.049	36	-28	22
R Midcingulate Area	1.66	6.73	<0.001	12	-12	44
R Superior Frontal Gyrus	2.11	6.44	< 0.001	24	0	54
L Superior Frontal Gyrus	1.26	5.94	0.001	-22	0	54
**Office > City (two-tailed)**						
R Precuneus	101.80	9.48	< 0.001	6	-56	36
R Precentral Gyrus	1.86	6.45	< 0.001	24	-26	66
L Middle Frontal Gyrus	4.02	6.42	< 0.001	-32	30	48
R Cerebellum 1	2.39	6.12	< 0.001	46	-62	-26
L Precentral Gyrus	1.18	5.82	0.002	-22	-28	62
L Cerebellum 6	1.06	5.51	0.003	-22	-70	-22
L Paracentral Lobule	0.82	4.92	0.013	-6	-12	68
L Superior Frontal Sulcus	0.93	4.44	0.007	-14	30	54
**Positive correlation with normalized bias**						
R Precentral gyrus	0.90	4.88	0.002	38	2	30
L Precentral gyrus	0.71	4.86	0.006	-48	0	54
L Supplementary motor area	0.82	4.53	0.003	0	0	64
R Superior Occipital Gyrus	0.48	4.03	0.041	24	-64	46
**Negative correlation with normalized bias**						
L Angular Gyrus	1.46	5.54	< 0.001	-40	-64	44
L Middle Frontal Gyrus	1.66	5.00	< 0.001	-2	-44	30
L Posterior Cingulate	0.62	4.96	0.013	-28	24	56
