# Supplementary material for: Trial-by-trial predictions of subjective time from human brain activity
Source: PLoS Comput Biol. 2022 Jul 7;18(7):e1010223. doi: 10.1371/journal.pcbi.1010223 (PMC9262235; doi:10.1371/journal.pcbi.1010223)
Supplement: Attachment — Submitted filename: time_as_events Response to reviewers .pdf [file pcbi.1010223.s012.pdf]

## Response to reviewers

First, we would like to thank both reviewers and the editors for their time and effort in considering our submission. We appreciate that the reviewers have taken the time to read our submission in depth and provide relevant and important comments and criticisms. We hope that our following point-by-point responses clarify the issues identified by the reviewers and thank them again for their contributions to improving our work.

We have pasted reviewer comments in blue, our responses are written in black, *quotes from the manuscript are italicised, and changes to the manuscript text are in red italics.* As well as responding to the specific reviewer points, we have made additional minor clarificatory changes to the manuscript throughout, noted using track changes.

### Reviewer #1:

Sherman et al provide a generally strong study of how the perceived duration of natural scene videos is correlated with the activity of the early visual cortex (measured with fMRI) and deep learning neural networks. Most importantly, they find that trial-by-trial variations in perceived duration follow the activity of early visual cortex. This finding supports the view that perception of a sensory stimulus’s duration reflects the activity of the sensory areas processing that stimulus rather than some centralised internal clock mechanisms that is often assumed to underlie time perception. Similar proposals are made by other recent studies that examine the changes in early visual cortex responses with event timing. The novelty of this study focusses on linking neural activity to perceived stimulus duration (rather than veridical stimulus duration) and using natural scenes like we experience in our daily lives (rather than the artificial, mathematically defined but easily controlled stimuli typically used in psychophysics).

Overall, I feel that the experiments provide clear results and careful controls are used. However, the interpretation of these results is complex, because natural scenes are not easily controlled for stimulus properties. While some effort is made to test the possibility that low-level visual features of the scenes underlie the visual cortex responses, these control analyses do not at all follow the known response properties of the visual system. This can be seen as a major shortcoming because it is exactly the responses properties of the visual system that are the focus of this study. But I feel that major changes to these control analyses are beyond the scope of this study, and major further analyses are not needed. Instead the problem is that the discussion of the known response properties of the visual system is lacking and rather dismissive, while in fact there are very good existing models of visual response properties, how visual responses are affected by stimulus properties, and how time perception is related to stimulus properties and the responses of the visual system. These should be discussed properly, constructively, and with reference to current models the visual cortex’s response properties.

First, thank you for the encouraging remarks about our submission. We apologise that our position has come across as dismissive of the importance of the temporal properties of visual response across the perceptual processing hierarchy. We believe that this is a simple miscommunication which we have rectified, as we describe below (and with corresponding changes to the manuscript).

We address the reviewer's point about the video-based control analyses (as presented in Figure 8) in our next reply, and in response to the point about visual response properties: we have added a substantial exposition to the Discussion, discussing many of the papers recommended at the end of the reviewer's comments, and have also made further changes which we outline later in our response.

In the first section of Discussion we now discuss the potential importance of visual cortex areas that were not included in our models:

*"Our visual cortex ROIs were chosen in such a way that our hierarchy was a highly simplified, "toy model" of human ventral vision. Of course, we by no means assert that other regions in visual processing are unimportant for duration perception of visual stimuli (23). Correspondences between stimulus features and neural responses have been shown in regions we have not included, and work that attempts to map the neural responses to temporal properties of visual stimuli has revealed a broad range of temporally responsive regions across the perceptual processing hierarchy, including dorsal visual stream and frontoparietal areas (see e.g. (24)). The present study suggests that for the specific stimulus content we utilized (silent videos) the ROIs we selected were sufficient to predict subjective time, but of course future work could test whether model performance would substantially improve by expanding the set of regions included.*

*We compared two different metrics for determining change in processing dynamics – Euclidean distance and signed difference, and used dynamic thresholds to classify changes as salient events, with these thresholds allowed to vary across layers of the perceptual hierarchy. We could predict human biases in duration judgements when using both metrics, but could only predict the video class (office scene vs. city scene) from the signed difference. It is notable that even these crude measures are sufficient for mapping processing dynamics within perceptual hierarchies to subjective time. Future work could attempt to optimise the metrics and thresholds according to differences in the known response properties of different visual areas (25,26)."*

In Discussion, Linking sensory content and subjective duration, we previously wrote:

*"In our approach, the neural processes that are engaged in the processing of sensory content (putatively ventral stream vision in our human participants) are the same as those used to build estimates of time. In this way we provide an intuitive link between sensory content and subjective duration. Our conclusion is in support of the idea that time perception depends on distributed mechanisms (27), but that in each case subjective time is naturally linked to sensory content by virtue of being determined by those content-related processes.*

Instead, we now discuss known relationships between stimulus properties and subjective time (paragraph 1), how they relate to our model and results (paragraph 2), and how our approach might be expanded to better incorporate those known relationships (paragraph 3):

*“It is common for studies in the time perception field to use highly constrained stimuli such as luminance contrast discs, Gabors, or random dot fields. The high level of control over stimulation permitted by such stimuli allows these studies to identify one-to-one mappings between a specific stimulus feature (e.g. contrast intensity, temporal frequency, or even stimulus size) and time perception. For example, a presented stimulus is reported as longer in duration in a condition where it is larger in size than in a condition where it is smaller (29) – a simple mapping between the feature of stimulus size and the corresponding duration judgements. A large literature exists showing how recent visual exposure (30,31) or adaptation to temporal properties of a stimulus can influence subjective time judgements (32–34); that eye movements nearby brief stimuli can change the content of vision, and consequently perception of time (35–37); how eye movements during stimulation change time estimates (38); and how the precise temporal properties of a visual stimulus – temporal frequency or speed – relate to subjective duration reports (2,3,39). Previous work has also shown how different parts of the processing hierarchy respond differently to temporal properties of stimuli, such as whether stimuli were composed of transient or persistent stimulation (26). Further, recent work using direct mapping methods with simple, brief stimuli (intervals up to ~ 2 seconds) has made substantial progress in describing the complex hierarchy of neural responses to temporal properties of visual stimulation (24,40).*

*The existing time perception literature, as described above, has been successful in characterising the relationship between visual response properties and subjective time. Our study extends this work by taking two key differences in approach. First, we did not directly look for a correspondence between temporal properties of stimulation and neural responses reflecting those temporal properties (i.e. we didn’t look for neural regions that represent the temporal properties of the stimulation – ‘chronotopy’). Instead, we attempted to build a model that extracts relevant temporal properties from the neural activity associated with processing of perceptual content and produces estimates of duration based on that information – an indirect rather than direct mapping between the stimulus and neural response to stimulus on the one hand, and subjective duration on the other. The second key difference in our approach comes from our use of naturalistic scenes versus the simple, highly-controlled stimuli frequently used. Natural scenes contain a wide variety and combination of simple and complex features, some of which will be coded for in overlapping neural populations. These features vary in their combinations moment to moment, and so to build a successful model of time perception for natural stimulation it is necessary to consider their joint contributions. By using a measure based on extracted salient events across the perceptual processing hierarchy, our approach accomplishes this task without needing to fully characterise those joint contributions. We note that to do so would be a very difficult task indeed. While a complete understanding of how these features are identified, tracked and combined in cortex remains an important challenge for visual neuroscience, and progress has been made (as we review above), our approach is able to jointly accommodate the many*

*stimulus features that vary during natural stimulation in a simple fashion, without needing to identify and track each pertinent feature independently, and then combine them in such a way that respects their interdependence (e.g. contrast drifts during configural changes).*

*There are interesting opportunities for future work at a greater level of specificity, which could attempt to fully specify the correspondences between all simple features and subjective duration, as well as their combinations. If successful, this detailed approach would allow very strong predictions about the precise, moment-to-moment contribution of some specific stimulus feature (and neural response to that specific feature) to subjective time perception. However, as our model demonstrates, having this complete understanding is not necessary for successfully modelling human distortions in subjective time."*

In Discussion, Biological plausibility, we discuss known response properties of visual cortex:

*"Our data do not speak to the question of how perceptual processing is achieved by the brain, and our results do not depend on the answer, beyond some key, uncontroversial assumptions (e.g. hierarchical processing). Whether the classification network used here (AlexNet) is closely matched to biological vision in how it processes information is not relevant here (see (14–16)) because the algorithmic approach to estimating duration from network activity (in either AlexNet or estimated from BOLD in humans) produces outcomes consistent with the patterns seen in human subjective reports of time. Accordingly, we did not directly compare the predictions from the classification network and brain-based models. The crucial assumption is simply the existence of a hierarchical, specialized system for perceptual processing - the common interpretation of primate ventral visual stream (48–50). ~~Given this assumption, whatever the specific computational processes underlying perception for the human brain are, the dynamics of perceptual systems implementing those processes can be used to construct subjective duration.~~ Given this assumption, our model is compatible with a range of prominent theories on the specific computational processes that may contribute to how perception is achieved (predictive coding (51), population coding (52), Gibsonian affordances (53) etc.), and the mechanisms underlying how feature extraction/processing occurs (population receptive fields (54), feedback connections (55), surround suppression (56) etc). Our claim is simply that the dynamics of perceptual systems can be used to construct subjective duration, but is theory-neutral as to precisely which processes are most important for perception. This conclusion is best demonstrated by the fact that our model produced estimates consistent with subjective biases in human reports regardless of whether applied to activation patterns of AlexNet or to BOLD patterns recorded from human participants."*

Major:

Specifically, Figure 7A convincingly demonstrates the accumulation of neural responses to salient visual events predicts the trial-by-trial bias in perceived movie

duration. Figure 8A then shows that there is no relationship between the Euclidean distance between pixels and human perceived duration. The problem with this analysis is that even an undergraduate textbook would make clear that the visual system does not simply respond to changes in pixel colour, and there is a whole literature on what the visual system does respond to. So, while this is a control for the possibility that duration perception depends on low-level stimulus properties, it is not well founded in the literature and is essentially a straw man argument: "it is not the changes detectable in the videos themselves that is key, but the response of perceptual classification networks". This does nothing to rule out a role of properties of the videos that are understood to underlie visual responses, like contrast, spatial frequency, temporal frequency or the changes in these properties over space and time. I do not think these properties need to be analysed (my third comment will clarify why not), but they do need to be discussed when considering what underlies the responses of the visual system.

Together with the reviewer's previous comment, our understanding is that the reviewer suggests our control analyses for stimulus complexity are unconvincing or superfluous because they ignore the well-known response properties of visual processing.

To clarify, the analyses presented in Fig. 8 and surrounding discussion were not intended to be presented as a control for visual stimulus complexity in terms of visual system response properties, nor to mimic responses to the visual system, but primarily as a reply to previous reviewers who have suggested that the stimuli themselves, by virtue of containing different amounts of physical change, were confounding the results. That is to say, if we understood the position of these previous reviewers correctly, that because our naturalistic stimuli contain different physical properties (by virtue of being naturalistic), our model was only trivially reflecting these physical properties of the stimuli.

The Fig. 8 analyses clearly show that the basic physical properties of the stimuli can dissociate from the model duration estimates, invalidating this argument. To reinforce the point (and hopefully avoid further confusion), the purpose of the presented analyses was not as a control for visual system response properties, but rather as a counter to the idea that naturalistic stimuli are somehow confounded by their own composition as naturalistic and therefore can't be used to study subjective time (a position we find surprising but have encountered repeatedly). So, we can't agree that the argument is a strawman, but we do agree that the point is largely unnecessary to make. To mitigate this potential source of miscommunication, we have made several changes to the manuscript.

First, we have moved this section associated with Fig. 8 (now Fig. S6) to the supplementary materials.

Second, the related section title *Subjective time perception is based on perceptual processing of stimulation, not stimulus changes directly* has been changed to *Pixel-wise changes in stimulation are dissociable from both human and model-predicted report*.

Finally, we have revised the text associated with Fig S6 to the following to make the above points clearer:

*"These two results demonstrate that the basic stimulus properties (here, simplified as change in pixel intensity) can dissociate from behavioural response. This may seem to be a trivial point, but it is important for highlighting the need to consider internal responses (as indicated by brain activity, measured or modelled). We are not claiming that changes in low-level stimulus features are irrelevant for time perception – of course, much of perceptual processing will be stimulus-driven. However, we do not take the approach that perceptual experience of these visual scenes directly and precisely reflects the physical qualities of those scenes without change; rather, in order to explain perceptual experience we need to consider neural responses to stimulation (modelled or measured), and not just the stimulation itself. That our approach tracks changes in brain dynamics across a hierarchy of processing is a feature that allows the model to deal with time perception on ~~a natural scale~~ for natural scenes. It is not a confound to be eliminated by considering only video stimuli perfectly matched for their lowest-level properties (i.e, mean pixel-wise distance) – since doing so would render such stimuli uninformative about naturalistic perception."*

We reference the (now) supplementary analysis at the end of the Results section:

*"In none of the auditory or somatosensory layers were there more salient events when watching city than office videos (Fig. 5, middle and bottom rows). Further, our ability to predict subjective biases in duration does not trivially follow from differences in the videos themselves (e.g. more changes in city than office videos), because frame-by-frame changes in the videos are dissociable from both human and model-predicted biases (see supplementary results). Taken together, these results underline the specificity of visual cortex activity in predicting subjective time for silent videos."*

Our revised Discussion text, presented on p.2-4 here, further explains how we have expanded our discussion about visual response properties.

I do not think it is enough to say that natural scene movies vary in many complex ways, and so write as if these variations are better ignored when studying natural scene movie perception. I have a lot of trouble with the following statement: "By using a metric based on salient events across the perceptual processing hierarchy, our approach accomplishes this task, allowing us to model natural stimulation in a way where tracking only a single stimulus feature would fail (e.g. tracking only luminance contrast intensity in natural scenes would not predict time perception for scenes where little luminance change occurs; tracking only motion energy would not predict time perception for scenes where there is little movement)." I appreciate the value of a hierarchical network in finding these combinations, but we can't say that a model based on a single feature would fail unless we try. The authors do not try. This type of statement is found throughout, and comes across as very dismissive of previous studies that use this approach to reveal important things about time perception (see the list towards the end of my review). I encourage a more constructive attempt to reconcile the current findings with previous literature.

We apologise for being too brief in our explanation of this issue. We agree that much has been learned about time perception through studies which map from the response properties for single simple stimulus features to time (as we now discuss extensively in our previous responses, above, especially p.2-4). To clarify how and why our approach differs, we have added further explanation to the introduction, which now reads:

*"That disruptions in time experience (i) arise across these different levels of stimulus complexity and (ii) are based on internal properties of the perceiver (such as what they are attending to) suggests that an approach is required that considers what is common across the hierarchy of perceptual processing, not just at a single level~~, is required~~. By identifying a measure ~~metric~~ that captures what is common across these features and levels of complexity and basing a model of subjective duration on it, our goal is to accommodate and bridge the many previously established relationships between content and time. Further, while many studies have attempted to find a ~~one-to-one~~ mapping (usually in the form of a correlation or similar analyses) between single, simple stimulus features and time perception (e.g. speed or temporal frequency (2–4)), natural scenes contain varying proportions of any single feature and these proportions will vary over time. Therefore, ~~to model~~modelling subjective time perception on the scale of natural stimulation will ~~clearly~~ require an approach that jointly considers the contributions of these different features."*

Following your helpful advice, we have removed the following contentious statement from the Discussion:

*"…where tracking only a single stimulus feature would fail (e.g. tracking only luminance contrast intensity in natural scenes would not predict time perception for scenes where little luminance change occurs; tracking only motion energy would not predict time perception for scenes where there is little movement)."*

The new section of our discussion (already pasted above on p. 3 here) attempts to motivate our use of a hierarchical model more constructively:

*"The second key difference in our approach comes from our use of naturalistic scenes versus the simple, highly-controlled stimuli frequently used. Natural scenes contain a wide variety and combination of simple and complex features, some of which will be coded for in overlapping neural populations. These features vary in their combinations moment to moment, and so to build a successful model of time perception for natural stimulation it is necessary to consider their joint contributions. By using a measure based on extracted salient events across the perceptual processing hierarchy, our approach accomplishes this task without needing to fully characterise those joint contributions. We note that to do so would be a very difficult task indeed. While a complete understanding of how these features are identified, tracked and combined in cortex remains an important challenge for visual neuroscience, and progress has been made (as we review above), our approach is able to jointly accommodate the many stimulus features that vary during natural stimulation in a simple fashion, without needing to identify and track each pertinent feature independently, and then combine them in such a way that respects their interdependence (e.g. contrast drifts during configural changes).*

*There are interesting opportunities for future work at a greater level of specificity, which could attempt to fully specify the correspondences between all simple features and subjective duration, as well as their combinations. If successful, this detailed approach would allow very strong predictions about the precise, moment-to-moment contribution of some specific stimulus feature (and neural response to that specific feature) to subjective time perception. However, as our model demonstrates, having this complete understanding is not necessary for successfully modelling human distortions in subjective time."*

We have made further minor edits to the expression throughout, with the aim of taking a more constructive tone.

At the other extreme, the authors test the predictions of deep convolutional neural network models. Here they find that these models do predict early visual responses and movie duration perception.

Apologies if this was miscommunicated in the manuscript, but to clarify: we only tested whether the DNN can predict biases in human duration reports. We didn't test whether they can predict visual cortex BOLD responses, nor whether they produce similar predictions to those from models based on BOLD. There was no direct comparison between the DNN and the BOLD models. We rely on existing work to demonstrate the degree to which convolutional neural networks may or may not correspond with the response properties of visual cortex. We have changed the wording to emphasise this point and explain why we do not directly compare the DNN and BOLD models.

Discussion:

*"Our results were robust under a wide range of model parameter values (Fig. 7B,D,F), and, in combination with ~~results from the perceptual classification network model and~~ previous findings (21,22), support the idea that human time perception is based in the neural processes associated with processing the sensory ~~context in~~content from which time is being judged."*

and

*"Our data do not speak to the question of how perceptual processing is achieved by the brain, and our results do not depend on the answer, beyond some key, uncontroversial assumptions (e.g. hierarchical processing). Whether the classification network used here (AlexNet) is closely matched to biological vision in how it processes information is not relevant here (see (14–16)) because the algorithmic approach to estimating duration from network activity (in either AlexNet or estimated from BOLD in humans) produces outcomes consistent with the patterns seen in human subjective reports of time. Accordingly, we did not directly compare the predictions from the classification network and brain-based models."*

While such neural network models are often seen as a black box, actually we understand that early network layers filter image inputs to detect contrast at particular orientations and spatial frequencies while middle levels analyse how the

outputs of these filters change over the image. These are exactly the stimulus properties that determine how the visual system responds, so the current analyses strongly suggest that such mid-level stimulus features underlie that activation of the visual system and also duration perception. Again, I therefore don't think it is enough to say that neural network responses are complex, treat them as a black box, and so write as if known visual response properties can be ignored. Again, as a study of the response properties of the visual system, I feel it is very important to discuss what we already know about the response properties of the visual system and try to bring the results of this study into line with that framework, even if this might have to be rather speculative without conducting a lot of further controls that are not necessary for interpreting the current experiments.

We understand the reviewer's perspective here and if we were indeed focused on the precise nature of visual response properties across the processing hierarchy – and the precise mapping of all such features into subjective time - we would be invested in addressing these aspects. We acknowledge that the reviewer's work takes a version of this approach and provides very robust and interesting demonstrations of what can be learned by doing so. But to be clear, we don't treat the neural network we used as a black box because we are unaware of the specificity of hierarchical response properties in them - we are indeed aware of these features of the network. As noted in the Introduction, the similarities with primate vision in terms of hierarchical arrangement and organisation of response specificity are important features which motivate why we used the specific deep convolutional network architecture (AlexNet trained on images) that we did for this purpose, as opposed to some other architecture that may perform better on image classification but is less relatable to primate vision in these features.

We have clarified this in the introduction to reflect the importance of this:

*"~~While the~~The neural ~~classification~~ network used in the previous study provided a reasonable stand in for human visual processing, demonstrating at least some of the useful functional properties of human visual processing hierarchy, such as its hierarchical arrangement, specialization of layers for different features, and increasing complexity of representations at higher layers (14,15)~~, fully assessing the above proposal naturally~~. There is ongoing debate about the degree and nature of similarities between such networks and biological vision, though relationships between classification performance and degree of representational similarity with primate visual processing can be found (16). Nonetheless, a full assessment of the above proposal requires neural as well as behavioral evidence from human participants."*

We have also added the following to Discussion: Biological plausibility (also on p.4 of this response)

*"The crucial assumption is simply the existence of a hierarchical, specialized system for perceptual processing - the common interpretation of primate ventral visual stream (48–50). ~~Given this assumption, whatever the specific computational processes underlying perception for the human brain are, the dynamics of perceptual systems implementing those processes can be used to construct subjective duration.~~ Given this assumption, our model is compatible with a range of prominent theories*

*on the specific computational processes that may contribute to how perception is achieved (predictive coding (51), population coding (52), Gibsonian affordances (53) etc.), and the mechanisms underlying how feature extraction/processing occurs (population receptive fields (54), feedback connections (55), surround suppression (56) etc). Our claim is simply that the dynamics of perceptual systems can be used to construct subjective duration, but is theory-neutral as to precisely which processes are most important for perception. This conclusion is best demonstrated by the fact that our model produced estimates consistent with subjective biases in human reports regardless of whether applied to activation patterns of AlexNet or to BOLD patterns recorded from human participants."*

We would note that we similarly treat the response properties of the human neuroimaging data as a 'black box' – something that the reviewer has identified elsewhere in their review. We know that human visual processing is broadly hierarchical and responsive to different complexity of visual information across the hierarchy, as we identify in section *Reconstructing human-like duration reports from visual cortex BOLD* when justifying our design decisions:

*"To do this, we defined a three-layer visual hierarchy a priori predicted to be involved in processing of the silent videos (see Fig. 3 and supplementary methods). We selected regions such that lower layers reflect the ~~detection and classification~~processing of low-level features (e.g. edge detection in primary visual cortex; V1), and higher layers, object-related processing (e.g. lateral occipital cortex; LOC). For control analyses, analogous hierarchies were built for auditory cortex and somatosensory cortex (see Table S1). "*

But, as with the artificial network, beyond these broad features we don't address response properties more deeply. In both cases, the choice to not address specific response properties is deliberate. The reasons for this are hopefully already clear to the reviewer from our responses above regarding mapping between specific visual responses and specific, single stimulus features and on to subjective duration.

There is one aspect of the deep network model I find puzzling. The model used is a feedforward model trained for object recognition, so models the ventral visual stream processing still images. On the other hand, the brain areas that seem to be most involved in representing visual stimulus timing are in the dorsal stream and frontoparietal network (and the early visual system, which is common to both dorsal and ventral streams) (see Hayashi et al 2018, Protopapa et al 2019, Harvey et al 2020, cited below). It's fine to use AlexNet as an easily available, existing model, but some strange conclusions are drawn as a result of this choice. Notably: "the response of perceptual classification networks … allows us to predict human reports of subjective time". Indeed the network used is trained for classification, but there is no effort to demonstrate that training for classification is required or that the ventral stream is important in the neural responses seen. Instead, it seems just as likely that it is the early stages of the network are picking up on the relevant movie features and would do so in a generative network that has not been trained for classification at all. Therefore, I feel that conclusions based on the structure (hierarchical, convolutional, feedforward) or training (classification, object processing) of the specific network used are not supported because no other networks were tested. These conclusions

are not central to this study, but the role of classification is mentioned repeatedly in the discussion, and I don't see why.

Perhaps this relates to the ambiguity about models matching neural responses mentioned above. Hopefully our response there has helped clarify this. In any case, we would like to emphasise that in using the term classification, it was not our intention to make a strong or controversial claim. For example, we would be comfortable describing the process accomplished by an orientation-selective neuron as classification (presented orientation is a member of some set: yes/no). To mitigate this potential issue, except where we are describing AlexNet (which *is* a classification network), we have changed "perceptual classification" to "perceptual processing" throughout the manuscript.

With respect to the dorsal versus ventral stream point, we now include the following in our Discussion:

*"Our visual cortex ROIs were chosen in such a way that our hierarchy was a highly simplified, "toy model" of human ventral vision. Of course, we by no means assert that other regions in visual processing are unimportant for duration perception of visual stimuli (23). Correspondences between stimulus features and neural responses have been shown in regions we have not included, and work that attempts to map the neural responses to temporal properties of visual stimuli has revealed a broad range of temporally responsive regions across the perceptual processing hierarchy, including dorsal visual stream and frontoparietal areas (see e.g. (24)). The present study suggests that for the specific stimulus content we utilized (silent videos) the ROIs we selected were sufficient to predict subjective time, but of course future work could test whether model performance would substantially improve by expanding the set of regions included."*

I also feel it is important to discuss previous studies that have taken a carefully controlled approach to these questions using simplified stimuli to investigate how the brain responds to stimulus timing, how responses of the human visual system underlie time perception and how stimulus features affect time perception. Again, I feel that highly relevant previous work is being ignored here. The authors should make their own choices here, but I hope they find these suggestions useful:

Bruno, A., Ayhan, I. & Johnston, A. Changes in apparent duration follow shifts in perceptual timing. J. Vis. 15, 1–18 (2015).
Heron, J. et al. Duration channels mediate human time perception. Proc. R. Soc. B Biol. Sci. 279, 690–698 (2012).
Zhou, J., Benson, N. C., Kay, K. N. & Winawer, J. Compressive temporal summation in human visual cortex. J. Neurosci. 38, 691–709 (2018).
Morrone, M. C., Ross, J. & Burr, D. Saccadic eye movements cause compression of time as well as space. Nat. Neurosci. 8, 950–954 (2005).
Johnston, A., Arnold, D. H., & Nishida, S. (2006). Spatially localized distortions of event time. Current Biology, 16(5), 472–479.
Bueti, D., Bahrami, B. & Walsh, V. Sensory and association cortex in time perception. J. Cogn. Neurosci. 20, 1054–1062 (2008).

Stigliani, A., Jeska, B. & Grill-Spector, K. Encoding model of temporal processing in human visual cortex. Proc. Natl. Acad. Sci. U. S. A. 114, E11047–E11056 (2017).
Kanai, R., Paffen, C. L., Hogendoorn, H., & Verstraten, F. A. (2006). Time dilation in dynamic visual display. Journal of Vision, 6(12):8, 1421– 1430
(And a preprint from my own lab: https://www.researchsquare.com/article/rs-579436)

Some more references about the dorsal stream and frontoparietal areas in time processing:
Hayashi, M. J., van der Zwaag, W., Bueti, D., & Kanai, R.. Representations of time in human frontoparietal cortex. Commun. Biol. 1, 1-10 (2018).
Protopapa, F. et al. Chronotopic maps in human supplementary motor area. PLoS Biol. 17, 1–34 (2019).
Harvey, B. M., Dumoulin, S. O., Fracasso, A. & Paul, J. M. A network of topographic maps in human association cortex hierarchically transforms visual timing-selective responses. Curr. Biol. 30, 1424-1434.e6 (2020).

Thank you for the recommendations. We have incorporated a majority of these, and some others, into our new discussion section (pasted above on p. 2-4), in which we describe some of the known relations between stimulus features and time perception, and some of their neural correlates. Specifically, we now discuss papers including:

Harvey BM, Dumoulin SO, Fracasso A, Paul JM. A network of topographic maps in human association cortex hierarchically transforms visual timing-selective responses. Curr Biol. 2020;30(8):1424–34.

Zhou J, Benson NC, Kay KN, Winawer J. Compressive temporal summation in human visual cortex. J Neurosci. 2018;38(3):691–709.

Stigliani A, Jeska B, Grill-Spector K. Encoding model of temporal processing in human visual cortex. Proc Natl Acad Sci. 2017;114(51):E11047–56.

Kanai R, Dalmaijer ES, Sherman MT, Kawakita G, Paffen CLE. Larger Stimuli Require Longer Processing Time for Perception. Perception. 2017;46(5).

Ulrich R, Nitschke J, Rammsayer T. Perceived duration of expected and unexpected stimuli. Psychol Res. 2006 Mar;70(2):77–87.

Tse PU, Intriligator J, Rivest J, Cavanagh P. Attention and the subjective expansion of time. Percept Psychophys. 2004 Oct 1;66(7):1171–89.

Bruno A, Ayhan I, Johnston A. Changes in apparent duration follow shifts in perceptual timing. J Vis. 2015;15(6):2–2.

Johnston A, Arnold DH, Nishida S. Spatially localized distortions of event time. Curr Biol. 2006;16(5):472–9.

Heron J, Aaen-Stockdale C, Hotchkiss J, Roach NW, McGraw PV, Whitaker D. Duration channels mediate human time perception. Proc R Soc B Biol Sci. 2012;279(1729):690–8.

Terao M, Watanabe J, Yagi A, Nishida S. Reduction of stimulus visibility compresses apparent time intervals. Nat Neurosci. 2008 May;11(5):541–2.

Morrone MC, Ross J, Burr D. Saccadic eye movements cause compression of time as well as space. Nat Neurosci. 2005 Jul;8(7):950–4.

Yarrow K, Haggard P, Heal R, Brown P, Rothwell JC. Illusory perceptions of space and time preserve cross-saccadic perceptual continuity. Nature. 2001;414(6861):302–5.

Grossman S, Gueta C, Pesin S, Malach R, Landau AN. Where Does Time Go When You Blink? Psychol Sci. 2019 Jun 1;30(6):907–16.

Kaneko S, Murakami I. Perceived duration of visual motion increases with speed. J Vis. 2009 Jul 22;9(7):14.

Hendrikx E, Paul J, van Ackooij M, van der Stoep N, Harvey B. Derivation of visual timing-tuned neural responses from early visual stimulus representations. 2021;

Signed,
Ben Harvey

**Reviewer #2:**

The paper by Sherman et al uses a previously artificial network model to predict human duration estimates of dynamic visual scenes, and then applies the same network model logic to recordings of human BOLD (fMRI) in visual areas and auditory/ somatosensory control areas. The findings replicate the previously found overestimation of scenes during which more changes occurred, both in human behavior and network output. Furthermore, BOLD activity from visual cortices shows the same bias, but only when signed changes across frames are modeled, not absolute change. BOLD from the control areas allows to reconstruct clock duration very accurately, but not the specific biases between scene types. Overall, the authors interpret their findings as evidence for time estimates being based on the neural processing of changes in visual inputs.
The approach is very interesting the results are convincing, but also quite complex. The study was preregistered during recording, and the data and codes are available. As detailed below, some aspects need to be clarified and better explained.

First, we would like to thank the reviewer for their generally positive comments. We genuinely appreciate the close attention paid by the reviewer to our work here.

1) When reading the Abstract and Introduction, I did not understand whether the authors claimed to be able to reconstruct full duration estimates, or just the biases thereof. After reading the full paper, I understand it is both, but the distinction could be made clearer from the start, especially since the authors classify the predictions

of clock time as trivial in the text. (As I will detail below, I am not sure how to interpret the ability of the models to estimate clock duration so accurately.)

Apologies for the lack of clarity. We have attempted to clarify this point in the abstract:

*"We were able to reconstruct trial-by-trial biases in participants' duration reports, which reflect subjective experience of ~~time (rather than veridical clock time),~~duration, purely from salient events in their visual cortex BOLD activity. ~~This was not the case for~~By contrast, salient events in neither of two control regions ~~in~~– auditory and somatosensory cortex – were predictive of duration biases. These results held despite being able to (trivially) predict clock time from all three brain areas."*

In the Introduction, we now write:

*"Control models applied to auditory or somatosensory cortex could produce reasonable estimates of ~~veridical time (i.e.~~ clock time, but these models did not predict participants' subjective ~~reports~~ trial-by-trial biases. Our model is, to our knowledge, the first that can predict trial-by-trial biases in subjective duration ~~reports~~ purely from measured human brain activity during ongoing naturalistic stimulation."*

We hope that this is no longer ambiguous.

Some sentences in the abstract and intro use terms like 'reconstructing human subjective time estimates' (Abstract, last sentence), or 'find a one-to-one mapping' (l.15-17). However, the studies cited in this respect (references 2–4) only test for correlations between the number of (perceived) changes and subjective duration estimates, but do not reconstruct the duration estimates (largely influenced by clock duration) themselves.

Please make this distinction and the implications thereof clearer.

We apologise for any ambiguity here. We used 'one-to-one mapping' because, on our understanding, the purpose of testing for correlations is precisely *to* find one-to-one mappings (correlations are just one example of one-to-one mappings). We have removed this from the text and hope that our meaning is now clearer.

*"By identifying a measure ~~metric~~ that captures what is common across these features and levels of complexity and basing a model of subjective duration on it, our goal is to accommodate and bridge the many previously established relationships between content and time. Further, while many studies have attempted to find a ~~one-to-one~~ mapping (usually in the form of a correlation or similar analyses) between single, simple stimulus features and time perception (e.g. speed or temporal frequency (2–4)), natural scenes contain varying proportions of any single feature and these proportions will vary over time. Therefore, ~~to model~~modelling subjective time perception on the scale of natural stimulation will ~~clearly~~ require an approach that jointly considers the contributions of these different features."*

Our extensive changes to the Discussion section *Linking sensory content and subjective duration* (pasted on p.2-4 of this response) may also be helpful for clarifying what we mean.

2) I am surprised by the models' ability to reconstruct clock duration almost perfectly. For the human brain this is not a trivial task (providing a signal that changes over time is, but reading out a duration that correlates with clock duration from it is not). To which extent does this linear increase in duration estimates with clock time result from the introduction of a temporal metric through the discrete time steps used in the model? For instance, l. 174: "These 'change' values were standardized within-participant and compared to a criterion with exponential decay to classify the change value as a salient event or not, giving us the number of salient events detected by each layer for each video." The way I understand this, the underlying discretization in time (TR) introduces clock duration into this analysis. Is this the reason for the ability to reconstruct sequence duration so well?

The reviewer is correct: if you introduce a temporal metric (through clock time) then you will inevitably have a proxy for clock time. Our metric is a temporal one (cumulative sum of positive numbers), and yes, this is why we can reconstruct sequence duration so well.

However, beyond that, the nature of the temporal metric doesn't appear to be very important. First, consider that if you have a single step between start and end and then just want a summary of what was in between. Here it's the number of salient events, but it could be something like the trajectory length approach in state dependent network model approaches – you have still got a temporal metric. In clock time, you have allowed physical time to elapse and then checked the state of the system at some later point. This will always be the case with any approach (sun dial, astronomical observation, etc).

We have clarified this in the text (Results: Using Euclidean distance, estimation bias but not effects of scene type can be reconstructed from visual cortex BOLD):

*"These results show that all models could reproduce ~~veridical~~ clock time – the physical duration of the presented video~~. The~~ - and therefore that the support vector regression model successfully mapped accumulated events to durations in units of seconds. This means we could use the regression model to further generate predictions of subjective duration biases from salient events in BOLD. It is important to note that the reproduction of ~~veridical~~presented duration is trivial because, all else being equal, longer intervals will have more salient events~~. For~~ in the sensory cortex BOLD dynamics. Indeed, longer intervals will, on average, have a greater number any event, task-relevant or otherwise – heart beats, eye movements, planes taking off, etc - and so the cumulative sum of events will trivially correlate with physical duration. Another way to think about this ~~reason~~,is that taking any summary – the number of salient events, the length of a trajectory through a state space, etc. - between a start- and end-point is a temporal metric, and so will correlate with clock time. "*

With regards to the discretisation into TRs, the important point (which is difficult to address specifically with the fMRI data) is that it seems the time scale doesn't matter much at all, within reason. From another comment, it appears that the reviewer may be aware of the supplementary results from the Roseboom et al. (2019) paper. There, we show that you can change rate of updating or skip frames etc and everything will broadly stay the same. Here, the network model updates at 60 Hz (the video frame rate) and the fMRI model updates at the TR, which was 800 ms. So together with our 2019 results, it is clear that rate isn't important as long as there is *some* time step over which change is considered (even if irregular), and our fMRI data show that even as that step approaches 1s in clock time, it still works well enough. Moving through clock time is essential to consider subjective time, but beyond that basic aspect it isn't important. We used this premise as a basis to consider a purely subjective foundation of time in our other recent work (Fountas et al. 2020, biorXiv; https://doi.org/10.1101/2020.02.17.953133)

Finally, to the important point about how our models can reproduce clock time when human participants will struggle with this task. It is important to consider that the regression models – which map from the units of sensation ("salient events") to clock time (in seconds) – are trained on all trials and so have access to a large set of different numbers accumulated salient events and corresponding clock durations from which they generate their mapping. In other words, the model is specifically trained to transform the number of salient events onto clock time, which is why it is easily able to do so. With feedback, humans can certainly get rather good at reproducing clock time too (our participants, even without training, exhibited a correlation of ~.8 with clock time). Of course, in more natural cases (outside of the lab) we perform worse: the task is much harder, mappings are contextual, and feedback is sparse (where people even bother to incorporate it) and over long time scales. In short, by virtue of simultaneous access to all information about relationships between time sensation (salient events) and clock time, our models have an unfair advantage versus humans, who may well be doing a similar thing (regression), but usually under very different conditions.

What would be the outcome of the model (artificial and BOLD) for constant inputs or inputs with random noise?

The correlation with clock time would be the same. To illustrate: suppose we draw 10 numbers from a standard normal distribution (random noise). Z-scoring all of them, we'd expect 5% to be greater than 1.96 or less than -1.96, so 2/10. If instead we draw 1000 numbers we would get ~200. Even though the correlation with clock time would hold, we can't see how accumulated changes in random noise could predict human estimation *biases.* BOLD responses to video stimulation are certainly not random noise.

Can you give a better explanation and rationale of these aspects, notably the exponential decay of the saliency threshold and the regression on seconds?

We regressed onto seconds to combine accumulated events in the 3 hierarchical levels into a single metric on the same scale participants use to communicate their experienced duration (seconds). It is during this regression process that the model combines the number of salient events across all the levels.

We have finessed our explanation of this point in the network model Results:

*"Salient events were accumulated at each layer and converted to estimates of duration in seconds via multiple linear regression, by mapping the number of accumulated salient events to the ~~veridical,~~presented (clock time), not reported durations (Fig 2A). This placed network predictions and human reports onto the same scale (seconds), and means that the model is attempting to reproduce clock time duration based on the input, rather than the more trivial task of training the model to directly reproduce human estimates."*

And in the brain-based modelling Results:

*"We then used support vector regression with 10-fold cross-validation to predict ~~veridical~~the presented (i.e., clock time, not subjective/reported) video durations from accumulated salient events in layers 1, 2 and 3 for each perceptual hierarchy. This converted the accumulated salient events in ~~each layer~~the three layers to a model-predicted duration "report~~s~~" in seconds so that they could be compared with human reports that were also made in seconds. This step was only necessary for directly comparing model ~~and~~output with human reports – as can be seen in Fig. 5, accumulated salient events (defined according to Eq. 3) in visual cortex ~~distinguished~~already distinguish between video type prior to transformation into seconds."*

The purpose of the exponential decay is to accommodate natural variations in the amount of change over some time course.  Roseboom et al (2019) show that the network model can still produce human-like biases in duration judgements when the threshold is constant (i.e. has no decay). We chose the decaying threshold both because we were building on the 2019 paper, but also because we think it is more intuitive: thresholds should adjust to the environment and task.

We now express this in Methods, after introducing the threshold function:

*"We chose the same criterion function used in (9): while they found that constant (not decaying) thresholds can also produce human-like biases in duration estimates, the decaying threshold reflects the intuitive notion that classification of what is salient should adjust with fluctuations in the environment."*

And in the Results, when describing the network model results:

*"Then, separately for each network layer, each distance – or change in activation – was categorized as salient or not. Note that a salient ~~event~~change is not necessarily psychologically salient, nor even a salient change in the environment; it is simply a relatively extreme change in dynamics. ~~This~~Saliency categorization was achieved*

*using an attention threshold with exponential decay that simply determined whether the change in node activation (the Euclidean distance) was sufficiently large to be deemed salient (see Methods). By decaying from the starting point to its minimum point, the threshold can adapt to local periods with few extreme changes.* Following *models of episodic memory* ~~*these*~~*(18) moments of threshold crossing are hereafter called 'salient events' (see also Discussion section "Surprise", time perception, and episodic memory and (7)).* "

3) Estimation of normalized bias:
In the description (l.68), what does 'category' refer to, the duration category or the visual category (city, office)?

We apologise for being unclear. We meant duration category. This has now been clarified:

*"For each of the k trials in which the* ~~*veridical*~~*presented video duration was t, the bias on trial k was the report on trial k, minus the mean report for that duration* ~~*category*~~*, divided by the mean report for that* ~~*category*~~*duration:"*

What is the reasoning for dividing by the mean report per category and not by the clock duration? If the mean estimates for half of the scenes are biased (the busy city scenes), then the mean estimate also reflects this bias and the single trial bias is capturing deviations thereof. It appears a bit uncommon to quantify biases with respect to the individual mean, rather than with respect to clock duration.

Dividing by clock duration gives percentage error relative to clock time. Percentage error will have both accuracy and bias components, whereas normalising by mean report gives us a measure of bias alone: it's calculating deviation from the *participant's* mean, not the degree to which the participant's mean report corresponds to the true duration. This is important to us because, for our purposes, our paper is not concerned with measuring how well participants can map their experienced duration onto seconds; we wanted to measure how *relatively* long participants' experience was, indicated via reports, for a given video (thus specifically capturing bias).

We have clarified this in the Results section:

*"For each trial, reported durations (in seconds) were transformed into our main behavioural measure: participants' bias towards under- or over-reporting of duration. This was quantified using a (pre-registered) normalized bias measure. For each of the k trials in which the* ~~*veridical*~~*presented video duration was t, the bias on trial k was the report on trial k, minus the mean report for that duration* ~~*category*~~*, divided by the mean report for that* ~~*category*~~*duration:*

$$bias_{t_k} = \frac{x_{t_k} - \overline{x_t}}{\overline{x_t}} \quad (1)$$

*Positive/negative values mean that individual duration reports were over-/under-estimated relative to the participant's mean for a given ~~veridical~~presented video duration. Therefore, normalized bias here is a bias (not accuracy) measure that reflects participant specific response patterns that are independent of clock time. This measure was then carried forward for subsequent analysis.“*

This point is now also made in the introduction as well, albeit more indirectly (necessarily so at this stage of the manuscript):

*"However, in this more recent proposal, there is a strong distinction in that change is not considered only as a function of changes in the physical nature of the stimulus being presented to the observer, but rather change is considered in terms of how the perceptual processing hierarchy of the observer responds to the stimulation.*

*The advantage of taking this approach is that we can accommodate the critical distortions that distinguish subjective duration from veridical 'clock' time. This was shown by Roseboom and colleagues (9), who used a deep convolutional neural network that had been trained to classify different images as proxy for human visual processing."*

4) Visual analogue scale
Generally, it would help to add a statement that the VAS asked for estimates in seconds, which were then used in those units in further analyses. Given that the true durations were 8-24 seconds, the range depicted by the VAS is skewed. How were the ends of the scale determined? Could this have increased the underestimation of the longer durations here?

Because we normalised duration reports within duration categories (i.e. separately for the 8, 12, 16, 20 and 24 second videos), participants' tendency to underestimate longer durations could not affect our measure of subjective duration *bias*.

With regards to the skew in scale, while it is true that the range is positively skewed in linear space, in log space it is negatively skewed (log(40)-log(24) ~= 0.5, log(8) ~= 2). As the reviewer may be aware, there is a substantial and long-standing debate regarding how linearly dimensions of experience, including time, are treated internally. Nonetheless, we agree it is possible that the range increased the underestimation of longer durations. As the reviewer notes below, it is also possible that this is a regression to the mean effect.

In the Results section we now clarify that reports were made in seconds, and that this was our key measure of interest:

*"[participants]...made duration judgements on a visual analogue scale ranging from 0 to 40 seconds."*

And also in the Results section:

*"For each trial, reported durations (in seconds) were transformed into our main behavioural measure: participants' bias towards under- or over-reporting of duration. This was quantified using a (pre-registered) normalized bias measure. For each of the k trials in which the ~~veridical~~presented video duration was t, the bias on trial k was the report on trial k, minus the mean report for that duration ~~category~~, divided by the mean report for that ~~category~~duration:*

$$bias_{t_k} = \frac{x_{t_k} - \overline{x_t}}{\overline{x_t}} \quad (1)$$

*Positive/negative values mean that individual duration reports were over-/under-estimated relative to the participant's mean for a given ~~veridical~~presented video duration. Therefore, normalized bias here is a bias (not accuracy) measure that reflects participant specific response patterns that are independent of clock time. This measure was then carried forward for subsequent analysis."*

Where was the cursor placed initially?

The initial placement of the cursor was random on each trial. We have clarified this in the Methods (Procedure):

*"A key grip was placed in each hand, and participants moved a slider left and right using a key press with the corresponding hand. The initial position of the slider was randomised trial-by-trial."*

5) Figure 2: the model-produced estimates show the same underestimation of longer durations as the human estimates. Can this be directly linked to a smaller proportion of salient events in the longer videos? How would the model estimate a concatenated series of two of the 8-s videos? As 2x8, or as ~13, as for the 16s videos?
In humans, this underestimation is attributed to regression to the mean effects, and it occurs for empty intervals, i.e. in absence of external change (Jazayeri & Shadlen 2010, Petzschner et al., 2015). How do you explain this tendency in the model? I found an explanation of this aspect in the 2019 paper and its supplementary materials, but I think it should also be explained in this work.

In theory, the model should estimate two 8-sec videos in the same way as one 16-sec video, with one possible additional factor - it will depend to some degree on the precise sequence of video content because of the decaying threshold (as a simplification, all videos start with threshold at starting position, but at the 8 s mark may be in another position, increasing chances of finding a "salient event").

Regarding regression to the mean, given that the estimates are mapped from salient events to seconds via a regression method, it is indeed inevitable that at least some underestimation at longer durations will occur due to this. We now explain this in the Results:

*"As was the case with human behavior, and as expected, the artificial classification network-based model produced duration reports that were significantly correlated with the video duration $\rho(2329)$ = 0.73, p < 0.001 (Fig. 2A)~~, indicating that the dynamics of the classifier were strongly related to clock time~~. Like our human participants, the model underestimated longer durations. As explained in Roseboom et al (9), this 'regression to the mean' effect is likely a product of mapping "sensation" (here, the accumulated salient events) onto a scale for report (here, seconds)."*

6) The definition of saliency is not quite clear to me.
l. 228: "The reproduction of veridical duration is trivial because, all else being equal, longer intervals will have more salient events." Can you illustrate what would be the salient events in audition or somatosensory domain?

Salient events here are salient events *in BOLD changes* (not in the stimulus). So as for vision, in the auditory domain a salient event is a relatively large difference in auditory cortex BOLD relative to the previous timepoint (TR). The same applies to somatosensory cortex. Salient events in the environment that might cause salient events in the respective sensory areas might be, for example, hearing a loud sound, or feeling a person tap you on the shoulder unexpectedly.

We now make this point in the text:

Results: Reconstructing human-like duration reports from human cortex BOLD

*"Voxel-wise changes between each TR were calculated and then summed over all voxels in the layer, resulting in one value per TR and layer. These 'change' values were standardized (z-scored) within-participant and compared to a criterion with exponential decay (and pre-registered parameters) to classify the change value as a salient event or not, giving us the number of salient events 'detected' by each layer for each video. Just as salient auditory events would be expected to correspond to large changes in (layer-wise) visual cortical activity, salient auditory events would be expected to correspond to large changes in auditory cortex dynamics, and may be trigged by, for example, hearing (or possibly imagining) a loud sound, and similarly for somatosensory cortex. Note that psychologically salient events need not map to salient events in BOLD; see Discussion section Predictive processing as a potential mechanistic basis for time perception."*

And in Results: Estimates generated by an artificial network model are biased by scene type

*"Then, separately for each network layer, each distance – or change in activation – was categorized as salient or not. Note that a salient ~~event~~change is not necessarily psychologically salient, nor even a salient change in the environment; it is simply a relatively extreme change in dynamics."*

However, as noted, a mapping between psychological and model defined salience need not necessarily occur in this way. As we say in the Discussion:

*“We also emphasize that the way in which we use “salience” and “surprise” is only tangentially, if at all, related to the psychological phenomena of something being salient or surprising. Here, salience is defined in terms of difference between successive network states (see Eqs. 1 and 4). This means our notion of salience is close to a naïve prediction error (9); naïve because the “prediction” is simply the previous network state rather than part of a prediction-update cycle (see (22)). While previous studies have suggested that predictability (62) or apparent salience (63) can affect subjective time perception (64), descriptions of “salience” and related terms at this cognitive level are not necessarily related to descriptions at the mechanistic level at which our model is articulated.”*

7) l 80: l. BFH(0,10.5) — why 10.5 ?

We apologise for the omission: we set our prior as a half-normal with mean of 0 and SD of 10.5 to represent our prior of a normalised bias difference of 10.5 for city vs office scenes. This difference comes from Roseboom et al (2019), Nature Communications (see Fig 3g there):

*“Mean deviation of duration estimates relative to mean estimate by scene type for human participants (mean shown in **a**; City: 6.20 mean (1040 trials), Campus & outside: 2.01 mean (1170 trials), Office & cafe: −4.23 (2080 trials);”*

Our prior of 10.5 comes from subtracting the city mean from the office and café mean (6.20 - -4.23 ~= 10.5). This detail was included in our preregistration and now appears in the Results where we write:

*“As predicted, durations of city scenes were relatively over-estimated and office scenes under-estimated, $M_{diff} = 5.18 \pm 1.36$ (normalized bias, %), 95% CI [1.81, 8.65], $t_{39} = 3.09$, $p = 0.004$, $d = 0.50$, $BF_{H(0,10.5)} = 33.8$, confirming that natural scenes containing a higher density of salient events do indeed feel longer (Fig. 1C. Our pre-registered prior for the Bayes factor came from the difference of 10.5 found in (6), see Fig. 3g there).”*

8) Fig.4 is lacking some detail:
Second panel: what does the color scale of the matrixes depict? What does the abbreviation TR stand for? What is X? Please spell this out in the caption.
What does the vertical black line reflect in panel 3 – zero ? Panel 4 has no axes labels.

We have added this detail and hope the figure is clearer now:

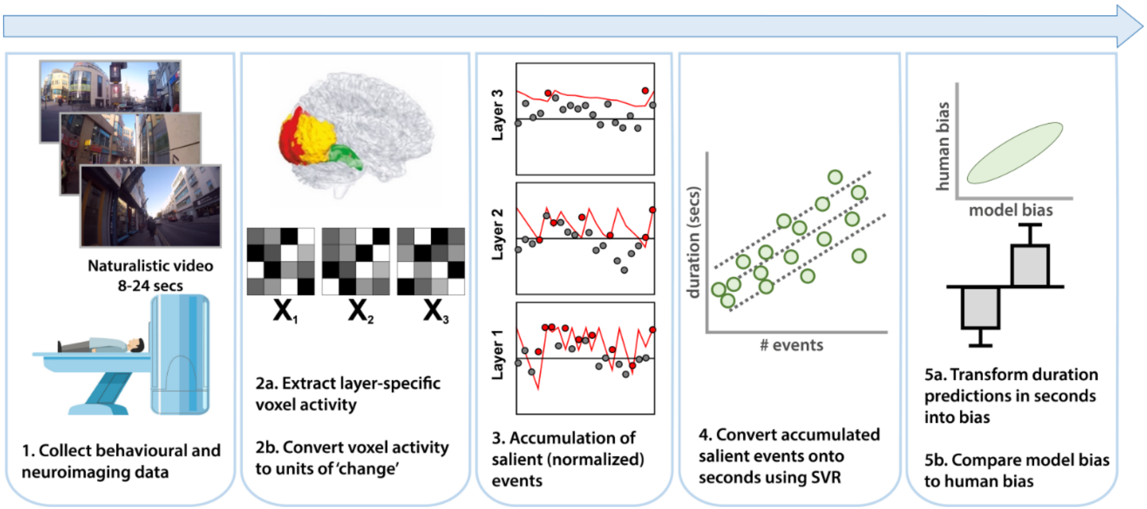


**Figure 4.** *Schematic of modelling analysis pipeline.* (1) Following data collection, (2a) voxel-wise ~~data~~BOLD amplitude was extracted and (2b) TR-by-TR (i.e. time point-by-time point, TR = repetition time) changes (Euclidean distance or signed difference) computed. The example given here is for the visual hierarchy, where each shaded matrix $X_k$ illustratively represents voxel-wise BOLD amplitudes (shaded squares) at each slice. ~~but t~~The same process was conducted for the auditory and somatosensory hierarchies (see Fig. 3 and Table S1 for different hierarchies). (3) Total change in the layer/ROI ~~over~~ at each TR was compared to a dynamic attention threshold (red line) that categorized events as salient (red dots) or not (grey dots). The black line represents 0. An event was classified as salient if it took an equal or higher value than the threshold. (4) Accumulated salient events were regressed onto seconds, (5a) predictions from the model were converted into normalised bias (5b) and compared across condition and with human behavior.

9) p.15: Non-significant p-values do not allow to conclude on the absence of an effect. Can you provide Bayes Factors to distinguish inconclusive from null effects (see e.g. Rouder 2009), especially for the ones were the absence of an effect is important for the interpretation (control areas)?

We agree completely. Thanks for bringing this to our attention.

For the pre-registered linear regressions (testing for associations between model and human biases), we took the beta coefficient from the visual model, b = 1.15, as a prior on the betas for the two control regressions (biases from the auditory and somatosensory models). The prior was modelled as a half-normal with a mean of 0 and SD of 1.15 (our prior):

*"Our ~~key,~~primary pre-registered hypothesis was that only the visual cortex model would be able to reproduce participants' duration biases. Supporting this, only the model trained on visual salient events significantly reproduced the ~~super-subject's estimation biases~~duration biases (calculated from the pooled, human "super-subject" data) trial-by-trial, $\beta_{2328} = 1.51$, $p = 0.015$; the models trained on salient events in auditory cortex, $\beta_{2328} = 0.87$, $p = 0.141$, and somatosensory cortex, $\beta_{2328} = 0.30$, $p = 0.339$, did not (Fig. S3). Using the visual cortex regression beta as our prior (20),*

*evidence for these control model results was insensitive (auditory: $BF_{H(0,1.51)}$ = 1.22, RR = (-∞, 7.56], somatosensory: $BF_{H(0,1.51)}$ = 0.60, RR = (-∞, 3.14]).”*

Evidence against an association between control model biases and behavioural biases was insensitive.

For the t-tests, examining whether biases derived from the visual, auditory and somatosensory brain-based models distinguished between office and city scenes, our prior was informed by the behavioural data. We set our prior as a uniform from a difference of 0 to a difference of 5.18 (the difference our participants demonstrated behaviourally). We chose a uniform rather than half-normal because it is likely that the brain-based biases would be smaller than the behavioural biases.

*“The control models did not reproduce the effect of video type either (auditory: $M_{diff}$ = -0.33 ±12.29, 95%CI = [-1.32, 0.67], $t_{2329}$ = -0.64, p = 0.522, d = -0.03, somatosensory: $M_{diff}$ = 0.16 ±13.09, 95%CI = [-1.23, 0.90], $t_{2329}$ = -0.30, p = 0.762, d = -0.01, see Fig. S2G-H). Setting a uniform prior between 0 and our behavioural effect of scene type (5.18%), Bayes factor analysis found evidence for the null in all three t-tests (visual: $BF_{U(0,5.18)}$ = 0.18, RR = [2.83, ∞), auditory: $BF_{U(0,5.18)}$ = 0.22, RR = [3.30, ∞), somatosensory: $BF_{U(0,5.18)}$ = 0.17, RR = [2.55, ∞)). Note that neither these t-tests nor the priors were ~~not~~ pre-registered.”*

We found evidence against a difference for all three brain-based models.

With regards to the exploratory (signed difference) analyses -

For the effect of scene type (office vs city), the visual cortex LMM had a fixed effect coefficient of 0.042. We used this as our prior for the LMMs tested on the auditory and somatosensory model predictions. Our prior was set as a half-normal (corresponding to a directional prediction) with mean 0 and SD = 0.042.

For the effect of human normalised bias on model normalised bias (the correlational LMM), the visual cortex LMM had a fixed effect coefficient of 0.018. We used this as our prior for the LMMs tested on the auditory and somatosensory model predictions. Our prior was set as a half-normal (corresponding to a directional prediction) with mean 0 and SD = 0.018.

Results are presented in the table below and show that we found evidence for the null for all controls except the somatosensory correlation, where the evidence was suggestive of the null.

| | **Bayes Factor** | **Robustness Region** |
|---|---|---|
| **Effect of scene type (office vs city)** | | |
| Auditory model | 0.159 | [0.020, inf] |
| Somatosensory model | 0.058 | [0.007, inf] |
| **Effect of model bias on human bias (correlation)** | | |
| Auditory model | 0.239 | [0.012, inf] |
| Somatosensory model | 0.460 | [-inf, 0.027] |

We have included this in the Results section:

*"While the model trained on accumulated visual cortex salient events reproduced patterns in human ~~behaviour~~biases, biases from exploratory models trained on auditory and somatosensory salient events did not: they neither discriminated video type (auditory: $M_{diff}$ = 0.36 $\pm$0.19, $\chi^2(1)$ = 0.43, p = 0.514, $BF_{H(0,0.02)}$ = 0.16, RR = [0.02, ∞], somatosensory: $M_{diff}$= 0.02 $\pm$0.21, $\chi^2(1)$ = 0.46, p = 0.499, $BF_{H(0,0.02)}$ = 0.06, RR = [0.01, ∞] see Fig 6E,F), nor predicted trial-wise human normalized bias (auditory: β = -0.003 $\pm$ 0.006, $\chi^2(1)$ = 0.20, p = 0.652, $BF_{H(0,0.04)}$ = 0.24, RR = [0.01, ∞], somatosensory: β = 0.002 $\pm$0.007, $\chi^2(1)$ = 0.11, p = 0.740, $BF_{H(0,0.04)}$ = 0.46, RR = [-∞, 0.03]* respectively, *Fig. 7C,E). Note that priors for the Bayes factors were not pre-registered and were set as the fixed-effect coefficients from the corresponding visual cortex LMMs."*

10) l.241-244: which estimation biases are meant here? This is a bit confusing, makes it easy to overlook that the model cannot reproduce the scene-type biases.

We are referring to the human normalised bias measure calculated after pooling across participants. We now clarify this in the text:

*"Our ~~key,~~primary pre-registered hypothesis was that only the visual cortex model would be able to reproduce participants' duration biases. Supporting this, only the model trained on visual salient events significantly reproduced the ~~super-subject's estimation biases~~duration biases (calculated from the pooled, human "super-subject" data) trial-by-trial…"*

11) Methods, p.31: please give more explanations for the decay function. Why does it vary across layers? What would happen if there was no change, or constant random noise?

Conceptually, the attention threshold varies across layers to reflect the fact that there should be fewer changes at higher than lower layers. This reflects the assumption of hierarchical processing motivating our modelling approach, with representations at higher layers being more abstracted. For example, if given a face with changing contrast then there will be lots of low level changes at but only few changes at higher layers, reflecting the onset and offset of the face but not the low-level changes in contrast. Accordingly, we set the threshold parameters at higher layers to be more conservative (have a higher maximum and minimum point). Practically, the precise parameters of the threshold were determined using pilot data (not included in our analysis), then pre-registered and used on our dataset here.

If we understand the second half of the question correctly, the reviewer is asking how the threshold would respond when the inputs are (a) no change or (b) constant random noise.

It would not be possible for there to be no change in the neural dynamics at all. However, if there was only *very little* change, then the threshold would only classify activity as salient once the threshold had sufficiently decayed. This is in contrast to a time period with large amounts of change, where a salient event might be classified earlier in the threshold's decay cycle. This is an advantage of the decaying threshold approach, though Roseboom et al (2019) in the supplementary materials show that the decay is not strictly necessary for predicting human estimation biases video frames.

In the case of constant random noise the threshold would respond in the same way as for any other input stream: the random noise stream (by "chance") will take locally extreme values that will be classified as salient. After this the threshold will reset, and decay again until it reaches another locally extreme point in the input signal.

We have added the following to the Methods to clarify this:

*"Then, for each trial, TR-by-TR categorization of $\Delta_{TR}$ was achieved by comparing against a criterion with exponential decay, corrupted by Gaussian noise $\varepsilon$:*

$$\vartheta_{TR} = ae^{-TR} + \varepsilon, \qquad \varepsilon \sim \mathcal{N}(0,0.05) \tag{4}$$

*We chose the same criterion function used in (9): while they found that constant (not decaying) thresholds can also produce human-like biases in duration estimates, the decaying threshold reflects the intuitive notion that classification of what is salient should adjust with fluctuations in the environment.*

*Only the parameter $a$ took different values in each layer (see Table S2): it took larger values at higher layers. In this way, the thresholds could accommodate different types of scene, e.g. scenes with more high-level configural changes, or scenes with more low-level changes. The criterion decayed with each TR until either an event was classified as salient or until the video finished, after each of which the criterion reset to its starting (i.e. maximal) point. Importantly, because the summed Euclidean distances $\Delta_{TR}$ were z-scored, the criterion has meaningful units corresponding to SDs above or below the mean."*

12) Fig 7., panels B, D, F: under some parameters, there seem to be significant associations between human and model bias, even for the somatosensory (low values for the upper bound), and auditory models (upper bound =2, lower bound = -2). Can you explain this?

For the auditory model, significant associations could be found when the upper bound is about 0, but this result is uninterpretable because such an upper bound is nonsensical: at its *most conservative* setting, the criterion classifies the mean amount of BOLD change as salient.

For the somatosensory model it is true that there were some small regions of the parameter space where significant associations were found, however it was far from widespread: there was a significant positive correlation between human and model biases in only 6% of the space, versus in 44% of the space for the visual cortex

model. Because these correlations are not robust to changes in the criterion parameters we conclude that they are either spurious, or an artefact e.g. from head motion or eye movements.

We now explain this in the Results:

*"The correlational results were also robust across a range of threshold parameters for the visual model (Fig. 7B). For the auditory model (Fig. 7D), positive correlations between human and model-predicted biases were found only at implausible parameter values (where the threshold's upper bound was the mean). For the somatosensory model (Fig. 7F), positive correlations were present in a small, localised region of the space indicating that those correlations were not robust to changes in threshold parameters, and likely spurious or artefactual (e.g., driven by head motion or eye movements)."*

13) Please discuss: What does it mean that the presented video duration can be predicted from visual, auditory, and somatosensory cortices? Do those regions (or the model) use the same clock?
How could a supra-modal decision be reached then? Could you speculate what would happen for videos with tones for instance?

We hope that the question of why we can predict video duration from all three sensory cortices has been answered in our responses to comment (2) above: because of the (temporal) metric we use, our ability to predict *presented* (i.e., veridical) video duration from all three sensory cortices tells us little about the neural basis of subjective time perception.

Flipping the reviewer's reasoning around here, consider the content we include in the Discussion section *Linking sensory content and subjective duration*. There we point out that only the activity related to visual perceptual processing is sufficient to model the subjective biases in time perception of our human participants, which supports the idea of, at least initially, modality independence in time estimation. This supports modality specific clocks, in that way of framing the problem. As we say in Discussion: Linking sensory content and subjective duration:

*" In our approach, the neural processes that are engaged in the processing of sensory content (~~perceptual classification processes~~putatively ventral stream vision in our human participants and for our stimuli) are the same as those used to build estimates of time. In this way we provide an intuitive link between sensory content and subjective duration. Our conclusion is in support of the idea that time perception depends on distributed mechanisms (28), but that in each case subjective time is naturally linked to sensory content by virtue of being determined by those content-related processes. ~~Our work provides an important step from previous studies on the topic.~~ "*

Given this initial modality independence, regarding supramodal time estimation, perhaps it is better to think of this as multimodal time estimation? If each processing modality contains the sufficient information to estimate time by itself, as suggested by our results, then an overall impression of time – built on the combination of this

information - could take several different forms. Perhaps the simplest speculation would be that it broadly follows decision rules from other multisensory cases and is consistent with Bayesian combination. Of interest to us, is that our model now provides a method from which to obtain modality specific estimates of duration to test against different potential models of combination. We say this in the Discussion, and have added this section to emphasise the point:

*"The model we present here is based on unimodal inputs, but for multisensory inputs we would predict that duration biases would be combined through some integration of accumulated salient events in modality-specific sensory cortices (e.g. for videos with sound, an integration of accumulated salient events in visual and auditory cortices). This may broadly follow what is understood about combination in other multisensory cases and may be consistent with Bayesian rules of combination (46,47)."*

Minor

Abstract: up to ~1 min / Methods: 8, 12, 16, 20 or 24 seconds.

Thank you for spotting this - the abstract now reads: *"Healthy human participants watched naturalistic, silent videos of up to ~~~1 minute~~24 seconds..."*

p.5, l. 49: participants' subjective reports

This now reads "*...but these models did not predict participants' subjective ~~reports~~ trial-by-trial biases."*

l.79: what is the unit of Mdiff – seconds? Please add it.

The numbers refer to normalised bias, which is % deviation from the participant's mean for that duration category. For brevity, we now write:

*"As predicted, durations of city scenes were relatively over-estimated and office scenes under-estimated, $M_{diff}$ = 5.18 +/- 1.36 (normalised bias, %)"*

Fig.3: add a color legend to the Figure

Fig. 3 has now been updated:

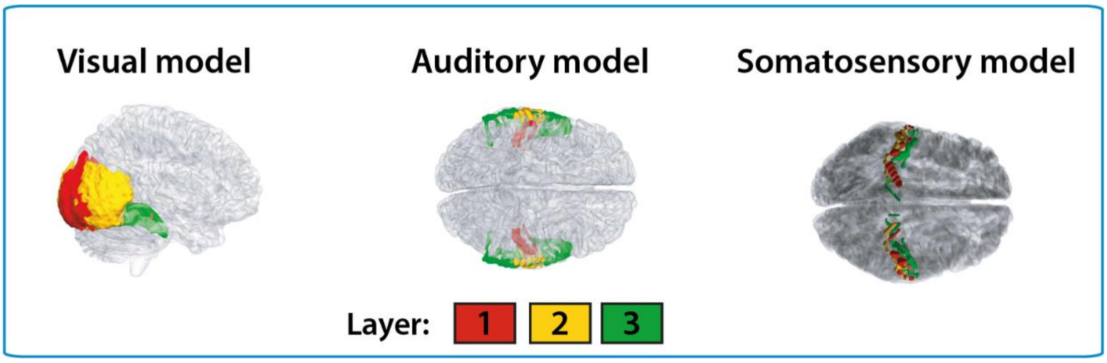


Fig.4: Panel numbers do not match the ones in the caption (3-5)

We apologise for the error. The figure caption has now been fixed:

**Figure 4.** *Schematic of modelling analysis pipeline.* (1) Following data collection, (2a) voxel-wise ~~data~~BOLD amplitude was extracted and (2b) TR-by-TR (i.e. time point-by-time point, TR = repetition time) changes (Euclidean distance or signed difference) computed. The example given here is for the visual hierarchy, where each shaded matrix $X_k$ illustratively represents voxel-wise BOLD amplitudes (shaded squares) at each slice. ~~but t~~The same process was conducted for the auditory and somatosensory hierarchies (see Fig. 3 and Table S1 for different hierarchies). (3) Total change in the layer/ROI ~~over~~ at each TR was compared to a dynamic attention threshold (red line) that categorized events as salient (red dots) or not (grey dots). The black line represents 0. An event was classified as salient if it took an equal or higher value than the threshold. (4) Accumulated salient events were regressed onto seconds, (5a) predictions from the model were converted into normalised bias (5b) and compared across condition and with human behavior.
