# Supplementary material for: Trial-by-trial predictions of subjective time from human brain activity
Source: PLoS Comput Biol. 2022 Jul 7;18(7):e1010223. doi: 10.1371/journal.pcbi.1010223 (PMC9262235; doi:10.1371/journal.pcbi.1010223)
Supplement: Attachment — Submitted filename: time_as_events_R2_ResponseToReviewers.docx [file pcbi.1010223.s013.docx]

**Response to reviewers**

We would like to thank both reviewers and the editors for their time in considering our resubmission. We hope that our following point-by-point responses to Reviewer 2 clarify the issues outstanding.

We have pasted reviewer comments in blue, our responses are written in black, *quotes from the manuscript are italicised, and changes to the manuscript text are in red italics.*

**Reviewer #1**

The authors have now addressed all of my comments, and I have no further concerns about this version. It's clear we take different viewpoints in places, but the neural representation of time is a new field and different researchers are welcome to have different views while we sort it all out. I thank the authors for their efforts, particularly in bringing their results into the context of vision science, by own background.

We thank the reviewer for their very helpful comments and considered response to our revision.

**Reviewer #2**

I would like to thank the authors for providing a thorough revision from which the paper has greatly benefitted.
Most of my previous points are solved (but the newly written parts have to be reread carefully for errors, see below). However, I still think that some parts of the paper can be misunderstood/ reflect an overstatement of the results: it should be made very clear that the model can estimate biases in human duration judgements, but does not provide inherent duration estimates as it relies on an externally induced metric of time. Furthermore, I think the behavioral measure needs to be better explained and justified.

Thank you to the reviewer for their careful reading of our revision. We have thoroughly explained and justified the normalised bias measure and hope it is now clearer – please see our response to comment 3 below.

With regards to the point about inherent duration estimates: if we understand correctly the reviewer is referring to the point that in order to transform salient events onto seconds we give the model the video duration in clock time. We now make this point explicit in the following places in the manuscript:

In section **Reconstructing human-like duration reports from visual cortex BOLD:**

*“We then used support vector regression with 10-fold cross-validation to predict the presented (i.e., clock time, not subjective/reported) video durations from accumulated salient events in layers 1, 2 and 3 for each perceptual hierarchy. This converted the accumulated salient events in the three layers to a model-predicted duration “report” in seconds so that they could be compared with human reports that were also made in seconds. It is important to point out by transforming from salient events to seconds in this way, the expression of estimates in seconds relies on an external metric of time - veridical video duration. Accordingly, the predicted durations in seconds by themselves are not the primary target of investigation. However, this regression step that involves an external metric of time was only necessary for directly comparing model output with human reports made in these units – as can be seen in Fig. 5, accumulated salient events (defined according to Eq. 3) in visual cortex already distinguish between video type prior to transformation into the units of this external metric of seconds.*

In the **Discussion:**

*“We compared two different metrics for determining change in processing dynamics – Euclidean distance and signed difference, and used dynamic thresholds to classify changes as salient events, with these thresholds allowed to vary across layers of the perceptual hierarchy. We could predict human biases in duration judgements when using both metrics, but could only predict the video class (office scene vs. city scene) from the signed difference. It is notable that even these crude measures are sufficient for mapping processing dynamics within perceptual hierarchies to subjective time. Future work could attempt to optimise the metrics and thresholds according to differences in the known response properties of different visual areas (25,26). Furthermore, while we regressed accumulated salient events onto video durations to express the predictions in seconds, future work could attempt to identify the neural substrates of this process of transforming an internal “representation” of duration into reportable units. Our model is agnostic as to how this occurs: the importance of perceptual processing here is in the construction of the basis for subjective time, and not in its final expression as a report in seconds.”*

1) Sentences like the following ones (and others throughout the manuscript) should be revised to coherently clearly state that the perceptual processing provides the basis for reconstructing duration estimation biases, but not the subjective time estimates as a whole.

It should also be made clear why the reproduction of clock duration by the model is trivial (currently mentioned in the abstract, but statements like the one below sound contradictory).

Abstract: "Our results reveal that the information arising during perceptual processing of a dynamic environment provides a sufficient basis for reconstructing human subjective time estimates."

l. 286: "To reinforce this point, it is because all of our models – visual cortex and the control models based on auditory or somatosensory cortex - do in fact provide reasonable estimates of clock time that our key analyses focus on reproducing the subjective biases present in the reports of human participants, since it is these biases that separate clock duration from subjective duration."

We now make the point about clock duration being trivial in **Results**, paragraph 2:

*“We reasoned that if subjective time is constructed from the accumulation of salient changes in the perceptual processing hierarchy, then we should be able to predict human over- or under-estimates of time from salient changes in visual cortex dynamics. We concentrated on over- or under-estimates of time rather than correspondence with clock time because the latter would be a particularly weak (and flawed) test of our hypothesis: the accumulation of any positive quantity – be it salient events in visual cortex dynamics while viewing a video or the number of births in Brazil while reading this paper – will increase with and therefore positively correlate with elapsed time.”*

We also mention this in **Discussion**, paragraph 2:

*“In this study, for which stimuli were silent videos, successful prediction was obtained only for models trained on salient events in visual cortex BOLD, and not for control models based on somatosensory or auditory cortex BOLD. While we could trivially reconstruct clock time from activity in all three sensory regions (because those regions exhibited dynamic neural activity that trivially reflects video duration), only the information extracted by the stimulus-relevant sensory model - the visual model - was related to subjective duration estimates (as reflected in relative over- or under-estimates of duration).”*

We now make this point again in **Discussion**, paragraph 3:

*“The reconstruction of clock time in all brain-based models was strong (correlations between model estimates and veridical video duration r > .92), and better than our human participants’ performance (correlation between participant estimates and veridical video duration r ~= .8). There are several reasons for this. First, as previously stated, the model predictions will trivially correlate with clock time more salient events will be accumulated as epoch length increases“*

In response to the reviewer’s point about reconstructing biases vs estimates, we now refer to “the *basis for* duration estimates” rather than just “duration estimates”. We hope that this makes clear that we do not suggest we have addressed the problem of how people make their duration reports (because we use the video durations to do that), but only the problem of how people generate a “representation” or “sense” of duration. We have made the following changes to the manuscript:

**Abstract**: *“Our results reveal that the information arising during perceptual processing of a dynamic environment provides a sufficient basis for reconstructing human subjective duration.”*

**Introduction**: *“In support of our proposal, we found that the model-based analysis could produce trial-by-trial predictions of participants’ subjective duration estimates based ~~only~~ on the dynamics of their multi-layer visual cortex BOLD while they watched silent videos.”*

**Estimates generated by an artificial network model are biased by scene type**: *“These results demonstrate that simply tracking the dynamics of a network trained for perceptual classification while it is exposed to natural scenes can produce the basis for human-like estimates of duration.”*

**Reconstructing human-like duration reports from visual cortex BOLD**: *“If we can predict trial-by-trial subjective duration only from participants’ BOLD responses in visual cortex (and not in other control regions), then we will have shown that the basis for human subjective duration judgements (when viewing natural visual scenes) can be constructed from brain activity associated with perceptual processing.”*

**Discussion**: *“We have shown that the basis for subjective estimates of duration can be constructed on a trial-by-trial basis from salient events in sensory cortex activity, where salient events are defined as relatively large changes in the neural responses to sensory stimulation across a perceptual hierarchy.”*

**Discussion - Linking sensory content to subjective duration:** “*In our approach, the neural processes that are engaged in the processing of sensory content (putatively ventral stream vision in our human participants and for our stimuli) are the same as those used to build the basis for estimates of time.”*

**Conclusions:** *“In summary, we provide evidence for an algorithmic account of duration perception, in which information sufficient for the basis of subjective time estimation can be obtained simply by tracking the dynamics of the relevant perceptual processing hierarchy.”*

2) The paragraph in the reply letter starting with "Finally, to the important point about how our models can reproduce clock time when human participants will struggle with this task…. " is very informative, and should be in the discussion of the paper.

We are glad it was informative. We have added a slightly edited version to the first section of **Discussion**:

*“The reconstruction of clock time in all brain-based models was strong (correlations between model estimates and veridical video duration r > .92), and better than our human participants’ performance (correlation between participant estimates and veridical video duration r ~= .8). There are several reasons for this. First, as previously stated, the model predictions will trivially correlate with clock time more salient events will be accumulated as epoch length increases. Second, we specifically trained these models to transform the number of salient events onto clock time (by mapping them to the video durations), and accordingly they do so very well. With feedback, humans can reproduce clock time well also – indeed, without training participants in this study exhibited a correlation of ~.8 with clock time. Outside of the lab, in more natural cases duration estimation may often be worse: the task is harder, mappings from experience to standard units of report are contextual, feedback may be sparse (and not necessarily even incorporated), and is received over long time scales. In short, by virtue of simultaneous access to all information about the relationships between time sensation (salient events) and clock time, our models have an unfair advantage versus humans, who could be doing similar computations (regression), but typically with less complete information.”*

3) The measure of bias in the analysis of the behavioral data: I am still a bit puzzled by the approach to quantify over-/ underestimation with respect to the participant's mean. Are there any references to justify this? Is the mean a meaningful parameter, i.e. are the distributions of duration reports roughly normally distributed (especially as they were collected on a VAS)?

The data are indeed (approximately) normally distributed. To illustrate this, we re-calculated normalised bias using the median instead of the mean. Then, for each participant separately, we correlated normalised bias calculated using the two summary metrics. When data are normally distributed, the mean is equal to the median. As can be seen from the histogram below all correlations were greater than 0.9, indicating that these two measures are effectively identical when applied to our data.

Taking reports relative to the participant’s mean (or median) is useful because it controls for any general tendencies to report high or low durations. By analogy, if I wanted to know whether my week’s grocery shop was particularly expensive I should compare it to my usual (mean/median) spend – which will be different from somebody else’s usual spend - and not to some objective metric.

Our justification for using this measure is (1) that it reflects trial-by-trial over-/under-estimates rather than accuracy, and (2) that it was used successfully in a previous paper on this topic (now in the manuscript as citation 10, Suarez-Pinilla et al). Accordingly we pre-registered our intention to use this measure for our analyses.

We have clarified these points, and also explained and justified the normalized bias measure in the Results section of the manuscript, as shown below:

*“For each trial, reported durations (in seconds) were transformed into our main behavioral measure: participants’ bias towards under- or over-reporting of duration. This was quantified using a ~~(~~pre-registered~~)~~ normalized bias measure that we have used previously (9). Normalized bias is a trial-by-trial measure that simply quantifies the percentage difference between each duration report and the participant’s mean report for the video duration category. In this way, it tells us whether reports were high or low as compared to typical reports by the participant made under comparable conditions.  For each of the k trials in which the presented video duration was t, the bias on trial k was the report on trial k, minus the mean report for that duration, divided by the mean report for that duration:*

$$\begin{aligned} {bias}_{t_{k}}=\frac{x_{t_{k}}-\overline{x_{t}}}{\overline{x_{t}}}\#\left( 1 \right) \end{aligned}$$

*Positive/negative values mean that individual duration reports were over-/under-estimated relative to the participant’s mean for a given presented video duration. For example, on a given trial that is physically 8 seconds long, if normalized bias is -0.5, then the report on that trial was 50% less than the average for 8 second videos. If normalized bias is 0 then the report was equal to the mean, and if it is 0.5 then the report was 50% greater than the mean. Note that normalized bias takes highly similar values when calculated using the median instead of mean (for all participants r > 0.9). Because normalized bias does not take the true video duration as an input it is a bias (not accuracy) measure that reflects participant specific response patterns that are independent of clock time. This measure was then carried forward for subsequent analysis.”*

4) Typos in the revision:

Thank you for spotting these and we apologise for the typos. We have now fixed them, as we show below.

"off any events"?

*Indeed, longer intervals will, on average, have a greater number
any event, task-relevant or otherwise – heart beats, eye movements, planes taking
off, etc - and so the cumulative sum of events will trivially correlate with physical
duration.*

This now reads: “Indeed, longer intervals will, on average, have a greater number of *any* event, task-relevant or otherwise – heart beats, eye movements, planes taking off, etc”

"Just as salient visual events " instead of auditory?

*Just as salient auditory events would be expected to correspond to large changes in (layer-wise) visual cortical activity, salient auditory events would be expected to correspond to large changes in auditory cortex dynamics, and may be trigged by, for example, hearing (or possibly imagining) a loud sound, and similarly for somatosensory cortex.*

This now reads: “Just as salient visual events would be expected to correspond to large changes in (layer-wise) visual cortical activity”
